# TRIM21 and Fc-engineered antibodies: decoding its complex antibody binding mode with implications for viral neutralization

**DOI:** 10.3389/fimmu.2024.1401471

**Published:** 2024-06-12

**Authors:** Johannes Reusch, Linda Elise Franken, Jakob Then, Philippe Ringler, Joachim Butzer, Thomas Juroschek, Christian Klein, Tilman Schlothauer, Laurent Larivière

**Affiliations:** ^1^ Roche Pharma Research and Early Development, Therapeutic Modalities, Roche Innovation Center Munich, Roche Diagnostics GmbH, Penzberg, Germany; ^2^ Roche Pharma Research and Early Development, Therapeutic Modalities, Roche Innovation Center Basel, F.Hoffmann-La Roche Ltd, Basel, Switzerland; ^3^ Biozentrum, University of Basel, Basel, Switzerland; ^4^ Roche Pharma Research and Early Development, Discovery Oncology, Roche Innovation Center Zurich, Roche Glycart AG, Schlieren, Switzerland

**Keywords:** TRIM21, therapeutic IgG, antibody Fc mutants, antibody mediated viral neutralization, binding kinetics, affinity, avidity, structure-function

## Abstract

TRIM21 is a pivotal effector in the immune system, orchestrating antibody-mediated responses and modulating immune signaling. In this comprehensive study, we focus on the interaction of TRIM21 with Fc engineered antibodies and subsequent implications for viral neutralization. Through a series of analytical techniques, including biosensor assays, mass photometry, and electron microscopy, along with structure predictions, we unravel the intricate mechanisms governing the interplay between TRIM21 and antibodies. Our investigations reveal that the TRIM21 capacity to recognize, bind, and facilitate the proteasomal degradation of antibody-coated viruses is critically dependent on the affinity and avidity interplay of its interactions with antibody Fc regions. We suggest a novel binding mechanism, where TRIM21 binding to one Fc site results in the detachment of PRYSPRY from the coiled-coil domain, enhancing mobility due to its flexible linker, thereby facilitating the engagement of the second site, resulting in avidity due to bivalent engagement. These findings shed light on the dual role of TRIM21 in antiviral immunity, both in recognizing and directing viruses for intracellular degradation, and demonstrate its potential for therapeutic exploitation. The study advances our understanding of intracellular immune responses and opens new avenues for the development of antiviral strategies and innovation in tailored effector functions designed to leverage TRIM21s unique binding mode.

## 1 Introduction

The tripartite motif-containing 21 (TRIM21) protein also known as Ro52 or RNF81, has emerged as a pivotal player in the dynamic interface between the adaptive and innate arms of the immune system (1). With its unique functions as effector and sensor occurring simultaneously, it triggers the antibody mediated degradation of viral capsids by the proteasome and activates innate immune signaling pathways (2–5). This unique intracellular receptor exhibits an ancient origin, and its importance in immune regulation and host defense has garnered significant attention and demands further exploration (6).

In healthy cells, a functional antibody is not capable of entering the cytosol since it neither has a mechanism to penetrate the cell membrane, nor can it exit the endosome during endocytosis (7, 8). However, it was reported that antibodies bound to incoming pathogens remain attached to viral capsids, even after endosomal escape, and are thus delivered into the cytosol (9). Here, TRIM21, an E3 ubiquitin ligase ubiquitously expressed in human cells, recognizes the Fc part of antibodies (associated to viruses) and directs the complex to the ubiquitin-proteasome system (UPS) for its degradation (10). This process is called antibody-dependent intracellular neutralization (ADIN). TRIM21 expression is drastically upregulated by interferon (IFN) stimulation thereby amplifying its neutralization activities (3). As an intracellular antibody Fc receptor, TRIM21 complements the traditional extracellular functions of antibodies by allowing them to function as immune regulators within cells and activate immune signaling pathways like the transcription factor NF-κB, AP-1 and IRF 3/5/7 (5, 11).

The Trim family of protein is characterized by a remarkably conserved N-terminal RBCC region (12). This region is composed of a really interesting new gene (RING) domain, one or two B-box domains, and a coiled-coil domain which represents the triumvirate giving the family its name (12–14). Trim proteins exhibit variations in their C-terminal regions, which are responsible for their specific functional attributes (15). For TRIM21, a C terminal PRYSPRY (PS) domain is responsible for target binding.

The RING domain functions as an E3 ubiquitin ligase, essential for recognizing and tagging proteins for degradation by attaching ubiquitin chains, signaling their breakdown by the proteasome (11, 16–18). The B-Box domain, specific to TRIM proteins, regulates TRIM21 by preventing auto-ubiquitination, thus regulating immune response activation (19–21). The coiled-coil domain (B-Box C-terminal; BBC) promotes dimerization of TRIM21 monomers (RBCC-PS), positioning RING domains at either end of an elongated antiparallel coiled coil domain, which is inactivated while the PRY-SPRY domain (B30.2) binds to IgG Fc, indicating its role in immune function (15).

In mammals, the TRIM21-IgG interaction is highly conserved, allowing the PRYSPRY domain to recognize IgGs from one species to various other species (22). Although TRIM21 has a lower affinity for the Fc region of IgA and IgM compared to IgG, targeting IgA still enables neutralization (23). As a homodimeric molecule, TRIM21 symmetrically binds to an IgG, engaging both Fc heavy chains simultaneously. This results in a 1:1 stoichiometry with the heterodimeric IgG (9). To date, only a single known apparent affinity/avidity dissociation constant (K_D,AVIDITY_) for dimeric TRIM21 binding to the IgG1 Fc wild type has been reported. This value is 600 pM, as measured by fluorescence anisotropy, while the kinetic rate parameters (k_ON_ and k_OFF_) remain undetermined. This interaction displays the strongest affinity observed in human IgG-Fc receptor interactions (9).

The binding affinity (K_D,AFFINITY_) between human IgG1 Fc WT and the recombinant human PRYSPRY domain has been measured to be in the range of 40 nM by isothermal titration calorimetry (ITC) to 200 nM applying surface plasmon resonance (SPR) technology, depending on technology and assay setup (9, 10, 24). The PRYSPRY domain (antibody Fc binding domain) interacts with both CH2 and CH3 domains, although the most substantial interaction is with the CH3 domain (24, 25). Like FcRn, the neonatal Fc receptor, TRIM21 interacts with the HNYH-motif where amino acids 429 – 436, especially H433, N434, H435, and Y436 of the CH3 Fc loop as well as I253 of CH2 are inserted into the PRYSPRY binding pocket (24, 26–30). FcRn prolongs the half-life of IgG by protecting it from lysosomal degradation and mediating its recycling back into the bloodstream. Unlike FcRn, TRIM21 – IgG interaction is reported to be largely pH-independent (from pH 5-8) but sensitive to salt. The ionic strength within cells is a critical determinant of biomolecular interactions, influencing protein activity, aggregation, and cellular processes essential for survival and proliferation (31, 32). Notably, the TRIM21-IgG interaction demonstrates a significant increase in affinity (5-fold) with reduced ionic strength, which is reflected by a slower off-rate when the salt concentration is decreased from 200 to 20 mM (22, 24). This sensitivity to ionic conditions demonstrates the importance of considering intracellular electrostatic environments when investigating the mechanism of action of TRIM21 and its role in immune complex degradation.

For the FcRn – IgG interaction there are reported mutations within the hotspot region, like YTE (M252Y/S254T/T256E), HH (T307H, N434H), which demonstrate increased FcRn binding, while mutating key residues into alanines (H310A, H433A and Y436A; AAA) completely abolishes it (33–41). In addition, there is a reported influence of the antibody variable domain (Fabs) on FcRn binding and pharmacokinetics, which has not been studied for TRIM21 (42, 43).

Recent findings highlight that TRIM21’s signaling requires higher activation than neutralization, which means that it is able to neutralize without initiating immune responses (2, 19). Engineered IgG1 variants with reduced TRIM21 binding still neutralize effectively but trigger less NF-κB signaling (10, 30). Mutations can significantly alter TRIM21 binding, with single mutations like H433A reducing activity, while others increase IgG1 Fc affinity up to 100-fold (10, 30). Slower dissociation rates from TRIM21 affect signaling more than neutralization, indicating possible low-level activity without an anti-viral state (10, 30, 44, 45). The distinction between effector and sensor activation thresholds allows for the effective clearance of minor viral threats without an extensive immune response.

Antiviral activity of TRIM21 has been demonstrated against a wide range of non-enveloped viruses, including adenovirus, Porcine reproductive and respiratory syndrome virus (PRRS), Japanese encephalitis virus (JEV), Hepatitis B virus (HBV) and rotavirus as well as certain bacteria (5, 9, 11, 46–51). It can interfere with infection by direct interaction with the viral proteins, as well as by regulating immune responses and by recognition of antibody decorated viral complexes (52). Upon the cytosolic entry of virus complexes opsonized by antibodies, the Fc portion of the antibodies is rapidly recognized by the catalytically inactive TRIM21 dimer. A proposed mechanism for the activation of viral degradation involves target-induced clustering (53). It is postulated that the intermolecular dimerization of the TRIM21 RING (between two or more TRIM21 dimers) domains triggers ubiquitination activity (54). This process potentially alleviates B-box inhibition of the RING domain, facilitates E2-Ubiquitin interaction, and constructs K63-linked ubiquitin chains on TRIM21 (19).

Previous research demonstrated that ADIN, which is triggered by catalytically active RING dimers, is remarkably efficient in IFN-stimulated mouse cells, requiring only 1.6 antibodies per virus to achieve effective neutralization (45). In contrast, IFN-stimulated human cells demand a higher antibody count, with 5 antibodies needed per virus for similar neutralization efficacy (45). It has been observed that the upregulation of TRIM21 can decrease the number of antibodies necessary for successful TRIM21-mediated neutralization (9, 45). In human cells that have not been stimulated by IFN, the ADIN process requires the recruitment of a greater number of TRIM21 molecules (53). Therefore, the nature of the target (oligomer) or the presence of multiple antibodies bound to the target could induce TRIM21 activation by promoting the clustering of multiple TRIM21 molecules in close proximity (53). TRIM21-antibody opsonization levels need to be above a certain threshold to enable RING dimerization, thus activating the innate neutralization pathway against the bound pathogen.

We sought to elucidate the detailed mechanism of action of the TRIM21-IgG interaction. To achieve this we generated several antibody Fc mutants, modifying TRIM21 binding. The used antibody variants express symmetrical, similar Fc heavy chains, or asymmetrical, varying heavy chains. We utilize a suite of complementary technologies to decipher TRIM21 binding. While it is established that single point mutations can modify PRYSPRY binding and that one TRIM21 homodimer interacts with one Fc heterodimer in a 1:1 stoichiometry with strong avidity, TRIM21 detailed mode of action remains unclear. This includes the precise nature of the natural dimer state, mutations that influence avidity, and determining how a synergistic interplay of TRIM21 mediated antibody variants bound to viruses leads to amplified avidity thereby influencing its mode of action.

In this study, we explore the mechanism by which the engagement of both heavy chains mediated via TRIM21 dimers takes place and examine the impact of Fc variants of antibodies on the balance between affinity and avidity, offering detailed insights into the mechanism of antibody binding. We demonstrate the occurrence of TRIM21 - anti-AAV antibody clustering on a biosensor, unveiling intricate binding dynamics. Gaining an in-depth knowledge of TRIM21 and its interactions with antibodies paves the way for innovative therapeutic approaches targeting infectious diseases, autoimmune conditions, and immune signaling pathways.

## 2 Results

### 2.1 Design of TRIM21 antibody biosensor assay configuration for dissecting its binding mode

**Figure 1:**
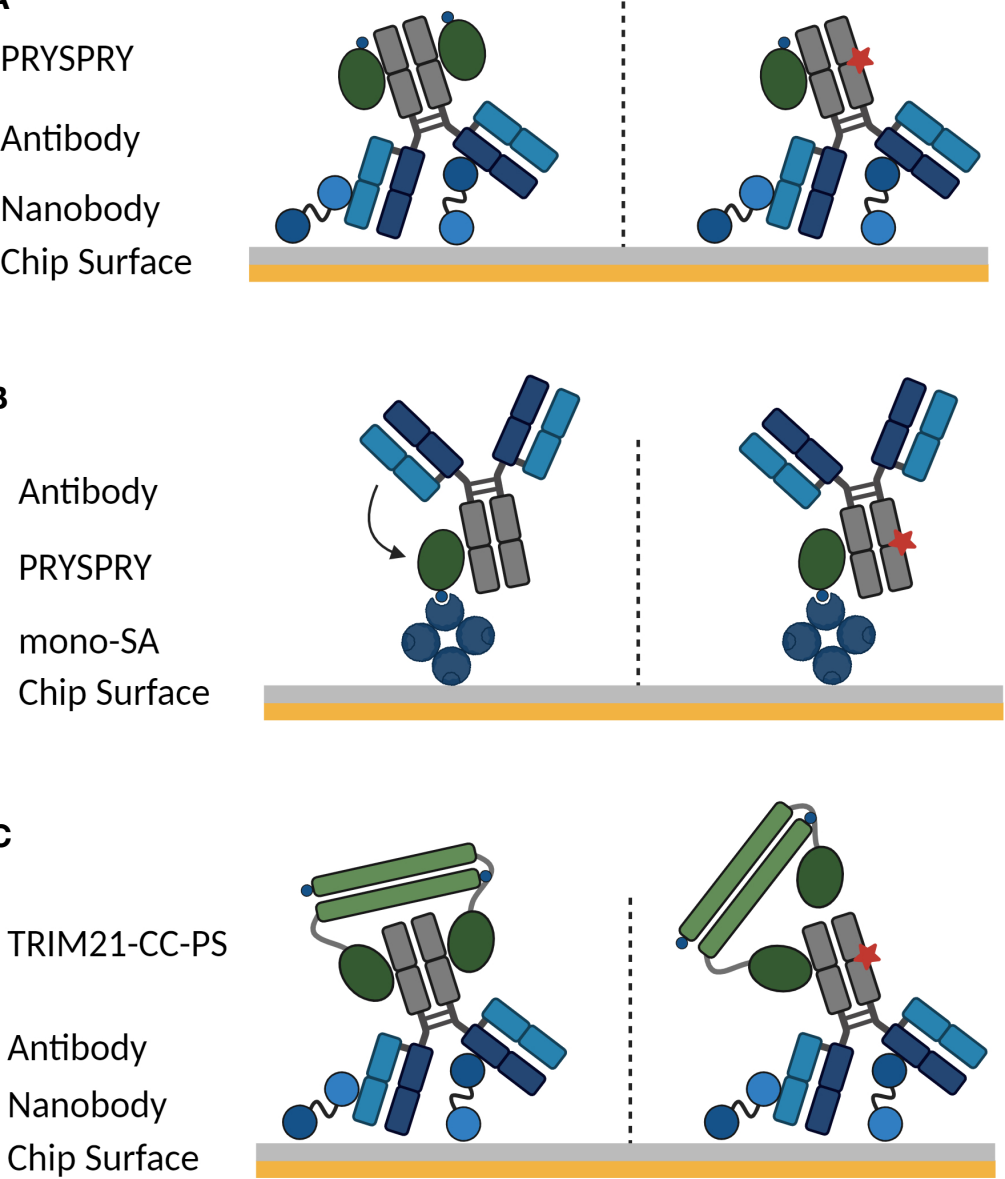
SPR assay orientations to characterize the interaction between TRIM21 and an antibody. Symmetrical and asymmetrical antibody Fc variants are investigated. In case of an asymmetrical Fc part, one Fc heavy chain contains a AAA mutation (schematically shown by red star), that completely abolishes TRIM21 binding. The used Fc variants and assay setups allow determining how Fc mutations influence the avidity-binding mode and dissecting avidity from affinity. **(A)** Antibody Fc variants are captured on the biosensor surface via an anti-Fab nanobody (vhh), Fc-only variants are coupled using standard amine coupling chemistry and cytokine Fc-Fusions are captured via anti-PGLALA F(ab’)2 fragment (55), while TRIM21 PRYSPRY domain is the analyte in solution (see Materials and Methods). Configuration **(B)** schematically shows the inverse to **(A)** while the PRYSPRY domain is captured via monovalent streptavidin. **(C)** To analyze the dimeric TRIM21 engagement of both IgG heavy chains, the antibody is captured via its Fab fragment, cytokine Fc-Fusions are captured via anti-PGLALA F(ab’)2 fragment (identical capture setup as in **(A)** and TRIM21-coiled-coil-PYRSPRY (TRIM21-CC-PS) is injected. Illustrations are created with BioRender.com.

To study the TRIM21 interactions in detail, there are two assay configurations possible on a (SPR) biosensor. Depending on the scientific question that should be addressed, either the antibody or TRIM21 is captured on the chip-surface (**Figure 1**). Furthermore, it is to be considered, that the bivalent antibody displays an Fc part with two binding moieties for TRIM21 and TRIM21 naturally forms a homodimer, which might cumulate in a more complex binding mode than a simple 1:1 interaction.

To investigate the TRIM21-IgG binding dynamics, we employed a strategic approach using two recombinant TRIM21 constructs (Sequence in Materials and Methods). We initially focused on the production of the PRYSPRY domain. This was essential to quantify the affinities of TRIM21 for various Fc-engineered antibodies, providing a clear assessment of how specific Fc modifications impact the binding affinity. By examining the interaction with the PRYSPRY domain alone, we could discern the direct effects of Fc engineering on the affinity component of the binding without the influence of avidity. Subsequently, we produced the TRIM21-CC-PS construct, comprising the PRYSPRY and coiled-coil domains to emulate the natural dimeric state of TRIM21. This construct is pivotal for investigating bivalent interactions with IgG antibodies, as it closely represents the native dimer and maintains the 1:1 stoichiometry of TRIM21 binding to both heavy chains of an IgG, as confirmed by a previous study, making it an ideal tool for our biosensor assays (9).

If the antibody (ligand) is captured on the surface under physiological conditions and the PRYSPRY domain (antibody Fc binding domain) (analyte) is in solution as shown in **Figure 1A**, the monovalent PRYSPRY domain binds to the Fc part exclusively in a monovalent fashion. Here, the mode of binding is independent from the ligand surface density. For the interacting pair of PRYSPRY and an antibody, this assay configuration exclusively probes the affinity mode, as the bivalent nature of the antibody Fc part is in this set-up irrelevant. Notably, this assay orientation allows the affinity characterization of various antibody Fc variants modulating the affinity towards the PRYSPRY domain. It also enables to investigate the stoichiometry for PRYSPRY and the dimeric Fc part, which we address with symmetrical and asymmetrical Fc variants. The latter abolishes TRIM21 binding for one Fc heavy chain.

The alternative orientation with PRYSPRY domain on the surface and the antibody in solution (**Figure 1B**) allows to investigate further attributes that describe the TRIM21-IgG interaction in more detail. As an example is the potential Fab contribution to TRIM21 binding efficiency as observed for FcRn binding (42, 43, 56–58). The ligand density in this assay setup is crucial. The antibody may interact with one or two PRYSPRY domains (ligand) depending on ligand density, by interlinking two domains that are close enough for simultaneous engagement (affinity & avidity). To exclude avidity binding modes we measured at low ligand density.

To analyze the TRIM21-CC-PS - antibody interactions, the antibody is captured on the sensor surface while TRIM21-CC-PS is injected (**Figure 1C**), enabling the observation of avidity effects from bivalent binding. Kinetic rate parameters for avidity, specifically k_ON_ and k_OFF_, remain uncharacterized for TRIM21. These kinetic parameters are more informative than the equilibrium dissociation constant (K_D_), offering a comprehensive view of binding kinetics. By determining k_ON_ and k_OFF_, we enhance our understanding of the interaction, which is essential for assessing the impact of Fc engineering on the TRIM21-antibody interaction. This detailed approach is critical for unraveling the effects of Fc engineering on both affinity and avidity, providing key insights for the development of therapeutic antibodies with enhanced specificity and improved therapeutic efficacy.

### 2.2 Antibody Fc engineerings to dissect affinity towards the PRYSPRY domain

**Figure 2:**
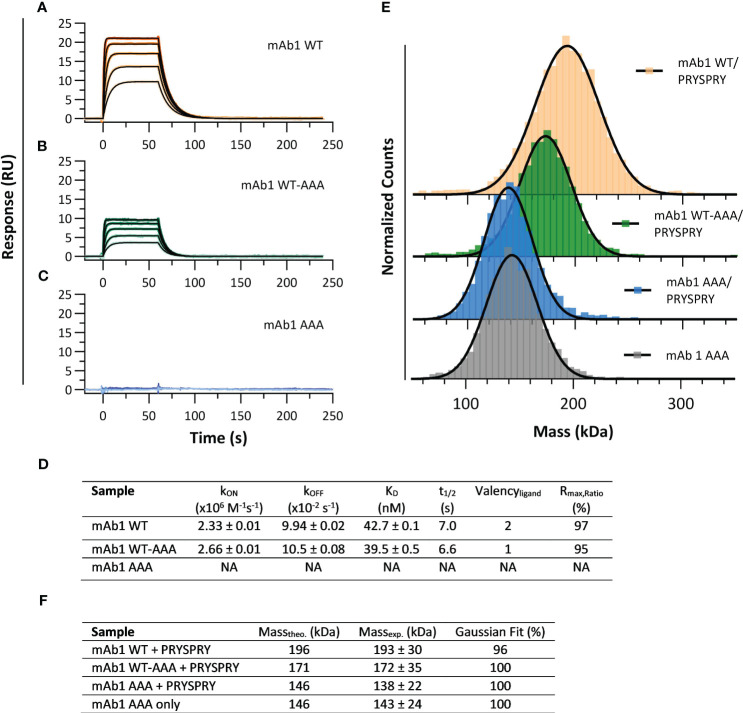
Interaction analysis of human IgG1 (mAb1) Fc variants and TRIM21 PRYSPRY domain. **(A–C)** showing sensorgrams (SPR data) where PRYSPRY was injected in five different concentrations as two-fold dilution series to immobilized mAb1 Fc variants (capture level approx. 60 RU). Each plot shows the measured raw data (colored gradient) and the global fit analysis as solid black lines. For immobilized mAb1 WT **(A)** and mAb1 WT-AAA **(B)** PRYSPRY was injected at 500 nM highest concentration and for mAb1 AAA **(C)** at 2000 nM. The sensorgrams show the affinity binding mode applying a mono-exponential fit model (Langmuir 1:1). The determined kinetic parameters are described in **(D)**. The k_ON_, k_OFF_ and K_D_ values are results from a global fit analysis ± fitting error. **(E, F)** show the complementary mass photometry (MP) data displaying a 2:1 binding stoichiometry confirming the SPR data. For the PRYSPRY - mAb1 WT complex, the data reveals a double bound state and for mAb1 WT-AAA a single bound state, while the control mAb1 AAA shows no binding at all. A Gaussian distribution model was used to analyze the MP data. For individual masses of the molecules, see SI Info **Supplementary Figure S1**.

To resolve the influence of Fc engineerings on the affinity for the TRIM21 PRYSPRY domain we used the assay setup shown in **Figure 1A**. To determine the kinetic rate parameters of the PRYSPRY - antibody interaction and its stoichiometry, a human IgG1 Fc WT (mAb1 WT) was compared with an asymmetrical variant mAb1 WT-AAA (H310A, H433A, Y436A). SPR binding kinetic of both constructs (**Figures 2A, B**) result in the same kinetic rate parameters. To confirm loss of binding in our AAA mutant, a double AAA mutant (mAb1 AAA) was used as control (**Figure 2C**). MAb1 WT and WT-AAA demonstrate an affinity dissociation constant (K_D,AFFINITY_, **Equation 1**) of 43 nM and 40 nM, respectively (**Figure 2D**, detailed parameters in SI **Supplementary Table S1**).

The R_max,Ratio_ (**Equations 2**, **3**) represents the ratio of experimental and theoretical maximal feasible SPR signal between a ligand - analyte couple, and provides information about the stoichiometry of an interaction (59). The interaction between mAb1 WT and mAb1 WT-AAA - PRYSPRY, with assumed binding sites of two and one, respectively, approaches 100% efficiency, leading to stoichiometries of 2:1 and 1:1, as shown in **Figure 2D**. This confirms that the IgG Fc binds two PRYSPRY domains, one at each of the CH2-CH3 interfaces. Additionally, the data shows that there is no effect of the first PRYSPRY binding event on the binding efficiency of the second monomer.

To confirm the findings from the SPR analysis we applied mass photometry (MP) for mAb1 WT/WT-AAA/AAA as a complementary method (**Figures 2E, F**). Considering the fast k_ON_ and k_OFF_ (**Figure 2D**) for the mAb1-PRYSPRY interaction, 200 nM mAb1 Fc variant were pre-incubated with 4000 nM PRYSPRY domain, and analyzed at a 1:250 dilution.

The non-binder variant mAb1 AAA with PRYSPRY (138 kDa) and without PRYSPRY domain (143 kDa), are in good agreement with each other and with the theoretical molecular weight (**Figure 2F**), demonstrating that PRYSPRY binding is abolished. An individual PRYSPRY domain appears at molecular weight of 32 kDa (Controls: **Supplementary Figure S1**). MAb1 WT + PRYSPRY gave a molecular weight of 193 kDa for the complex, which is in agreement with two PRYSPRY bound to one antibody (Gaussian Distribution Model), while mAb1 WT-AAA + PRYSPRY shows only the single bound state represented by 172 kDa.

**Figure 3:**
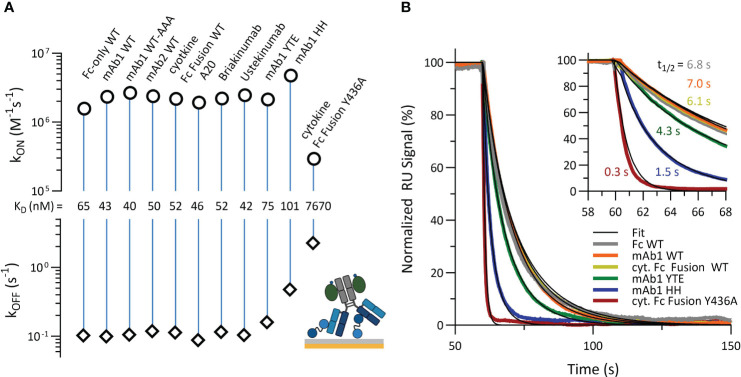
Kinetic characterization of TRIM21 PRYSPRY binding to immobilized antibody Fc variants and cytokine-Fc Fusion constructs, and Fc only variant (Raw data SI Info **Supplementary Figure S2**). Detailed SPR assay setup is described in materials and methods. **(A)** The Affinity Rate Scale Plot enables the kinetic comparison of several binding experiments at one glance. The association rate (k_ON_) and corresponding dissociation rate (k_OFF_) are juxtaposed in opposition, connected via a vertical line, representing the binding strength (affinity). The further apart both parameters (k_ON_ and k_OFF_) the stronger the interaction is. Compared to mAb1 Fc WT, the Fc variants YTE (M252Y, S254T, T256E), HH (T307H, N434H) and Y436A show decreased PRYSPRY affinity. The YTE affinity is 1.7-fold, HH 2.4-fold and Y436A 180-fold decreased. As shown in **(B)** the altered binding strength is mostly off rate driven, which becomes apparent in the overlay of normalized dissociation. The start of dissociation is normalized to 100%.

To further examine how PRYSPRY binding can be altered by antibody constructs and Fc engineerings, we characterized additional variants, applying the assay setup shown in **Figure 1A**. All measured antibody formats share an identical IgG1 Fc framework, with or without specific mutations within TRIM21 binding interface. mAb1 WT and mAb2 WT exhibit different electrostatic and hydrophobic Fab patches. Two additionally investigated constructs are Briakinumab and Ustekinumab. Briakinumab shows a large positively charged Fab region, which is absent in Ustekinumab, and has been shown to have different FcRn affinities (42, 60). A cytokine-Fc Fusion and a Fc-only (CH2-CH3) WT were also included along with the mAb2 WT knob-into-hole (KiH) variant, which has specific mutations in the CH2-CH3 region (details in materials & methods). To compare the determined affinities, the kinetic rate parameters are displayed in a kinetic rate scale plot (**Figure 3A**). Detailed sensorgrams and kinetic parameters are shown in SI Info **Supplementary Figure S2**.

In this assay orientation, the affinities of most antibody constructs for PRYSPRY are similar. Exempt are the Fc variants YTE (M252Y, S254T, T256E), HH (T307H, N434H) and Y436A, which contain Fc mutations at key residues in the binding interface for PRYSPRY. These variants have lower affinities for the PRYSPRY domain than WT, showing a sequence from highest to lowest strength of binding as follows: WT has the strongest affinity, followed by YTE, then HH, and Y436A has the weakest.

For YTE the effect is only off rate driven (1.6x faster compared to WT), while for HH and Y436A it is a combination of both on and off rates. In contrast to WT, HH shows a 5x faster off rate, while the on rate is 2x faster, compensating for a significantly weaker affinity (K_D,AFFINITY_) compared to the YTE variant. For the Y436A variant both rates are strongly decreased (8x on rate and 23x off rate compared to WT). Overlaying the normalized dissociation phases (**Figure 3B**) shows the significant effect of Fc engineering (t_1/2_: 7.0 s for WT vs 0.3 s for Y436A).

Here, we showed that our assay setup (**Figure 1A**) is suitable to determine the pure, undisturbed affinity of the PRYSPRY-IgG interaction. We found a 180-fold reduced affinity for Y436A compared to WT. The SPR and MP results reveal a PRYSPRY-IgG stoichiometry of 2:1 without any binding cooperativity. The chosen assay setup does not reveal an additional contribution of Fab binding, as such we apply the reversed assay orientation to address this topic in the following section.

### 2.3 Impact of antibody variable domain on TRIM21 PRYSPRY binding affinity

**Figure 4:**
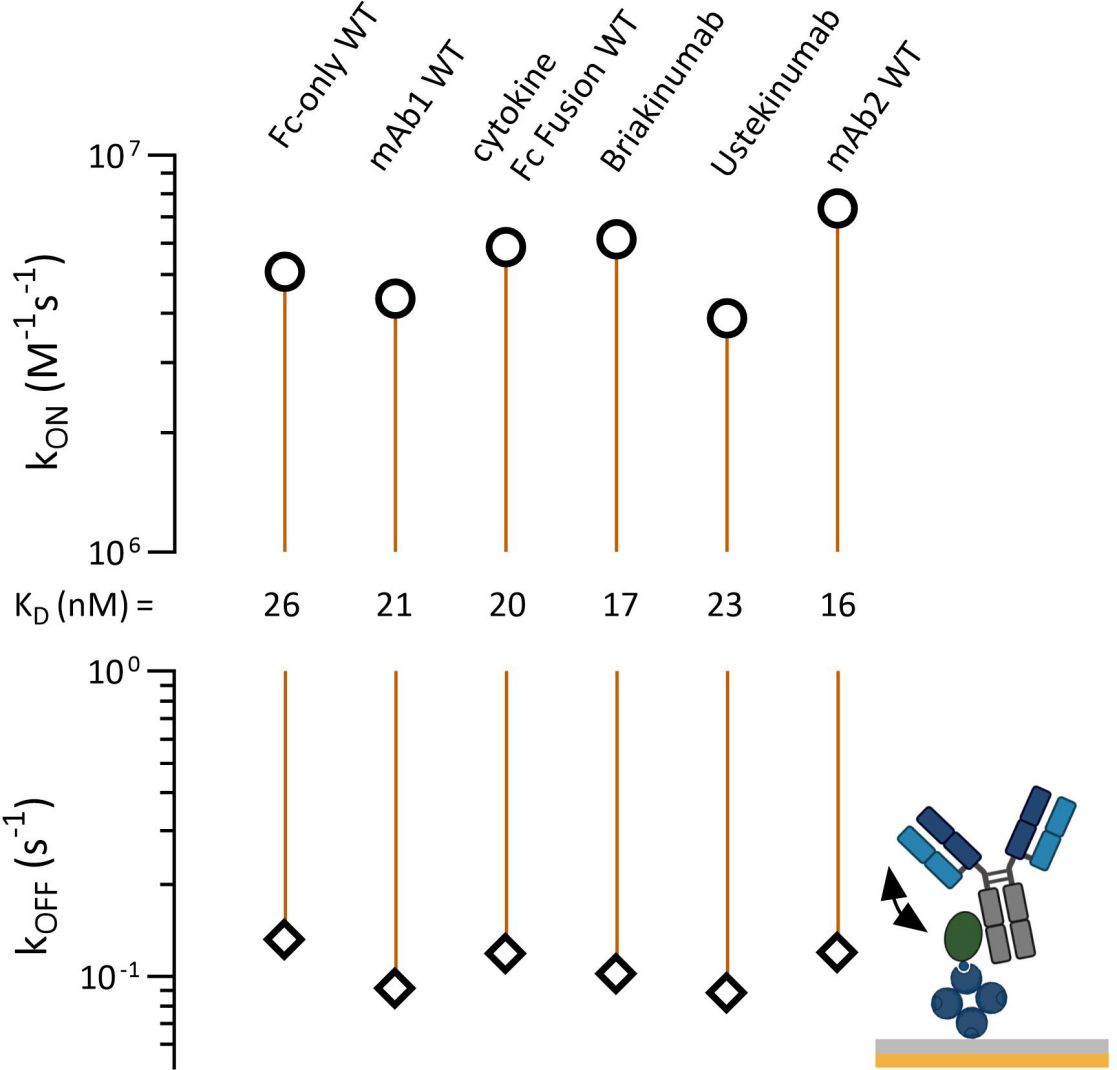
Affinity rate scale plot for captured TRIM21 PRYSPRY domain (ligand) and antibody (human IgG1) variable domain variants or antigen fusion constructs in solution (analyte). The injected constructs have different Fab regions but share the same Fc region. This allows the investigation of a potential Fab contribution to the PRYSPRY binding. All constructs were analyzed by applying a simple 1:1 Langmuir fit. The analyzed antibody variants do not show any Fab contribution. Notably, there is a faster on-rate (2x) for all constructs when compared to the reverse assay setup up (PRYSPRY as analyte). Raw data is shown in SI Info **Supplementary Figure S3**.

FcRn and TRIM21 share an overlapping CH2-CH3 binding interface. Antibodies with an identical Fc part but different Fab domains bind differently to FcRn, showing different affinities and pharmacokinetic properties (42). To uncover a potential PRYSPRY-IgG Fab contribution, we reversed the assay setup while allowing the Fab arms to move freely in solution (**Figures 1B**, **4**, detailed data in SI Info **Supplementary Figure S3**). To prevent intermolecular interactions where a single antibody binds to two adjacent PRYSPRY domains, we used low PRYSPRY densities (detailed in Materials and Methods, Section 7.5). The surface density of the PRYSPRY domain was empirically optimized until the data could be accurately analyzed using a monophasic fit model. This model reflects the affinity-binding mode, ensuring that no avidity effects arise from interactions with two adjacent PRYSPRY domains.

The reverse assay orientation reveals a two to three-fold increase in binding strength. This effect is primarily driven by on-rate changes, not influenced by Fab regions, as evidenced by same outcomes for the control (Fc-only WT), which lacks a Fab. The affinities across the characterized antibody panel remain consistent with each other, confirming the absence of any measurable Fab effect on the Fc-PRYSPRY interaction.

### 2.4 The dimeric state of TRIM21-CC-PS

**Figure 5:**
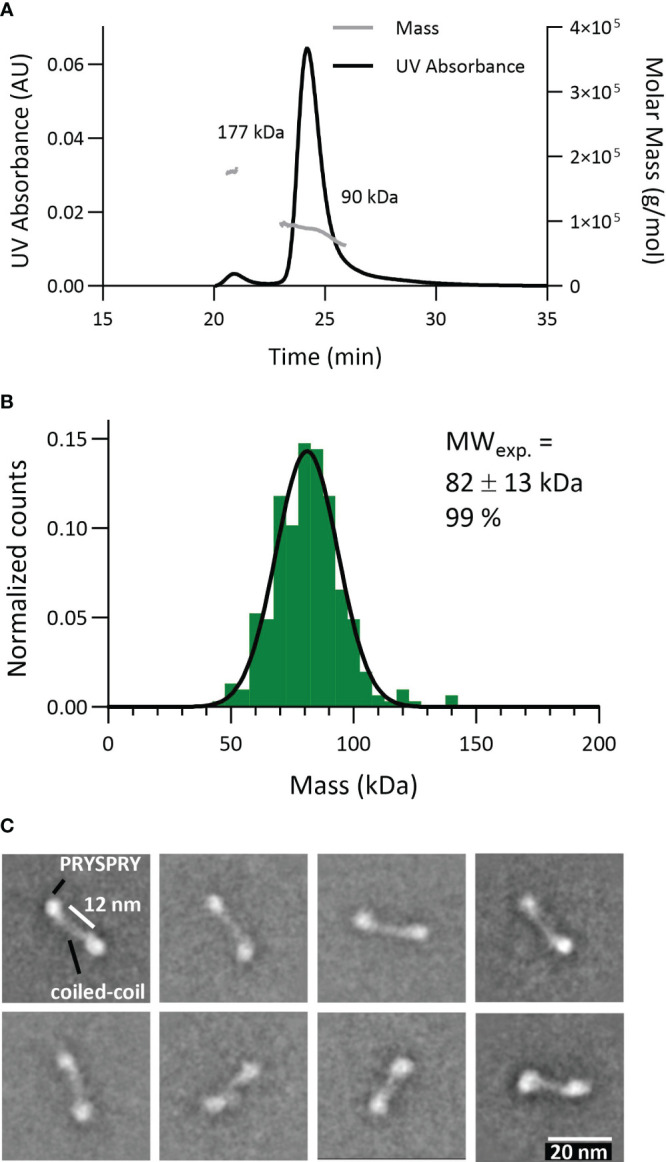
Characterization of the TRIM21 dimeric nature (TRIM21-CC-PS) applying different technologies. **(A)** SEC-MALS data reveals 94% TRIM21-CC-PS dimer (90 kDa). **(B)** Mass photometry technology shows 99% TRIM21-CC-PS with 82 kDa. **(C)** Selection of EM 2D classes confirming TRIM21-CC-PS dimers. The coiled coil domains facilitate dimerization whereas the C-terminal PRYSPRY domains are placed at the opposite end of each coiled-coil domain.

To probe the dimeric engagement of both IgG heavy changes simultaneously, we first characterized the construct TRIM21-CC-PS (**Figure 5**). The expected theoretical molecular weight of this dimeric construct is 86 kDa. Applying SEC-MALS revealed a mass of approximately 90 kDa for 94% of the particles (4% showed 177kDa corresponding to a tetramer) (**Figure 5A**). MP gave 99% of particles at a weight of 82 ± 13 kDa (**Figure 5B**). Electron Microscopy analysis revealed that the PRYSPRY domains are facing away from each other with the coiled-coil domains in between mediating dimerization. Shown 2D classes indicate a slight variation in the position of the PRYSPRY domains with respect to each other (**Figure 5C**). Whether these variations are caused by the limitations of the applied technique or are biologically relevant has to be investigated.

Here, we confirmed the dimeric nature of TRIM21-CC-PS with three techniques. Surprisingly, the EM data show that the coiled-coil domain is stretched and the distance between the two PRYSPRY domains is extended. The fact that we were able to resolve the shape of the coiled-coil is an indication that in its dimeric shape TRIM21 is relatively limited in its flexibility.

### 2.5 TRIM21-CC-PS – antibody Fc variant Interaction by mass photometry and electron microscopy

**Figure 6:**
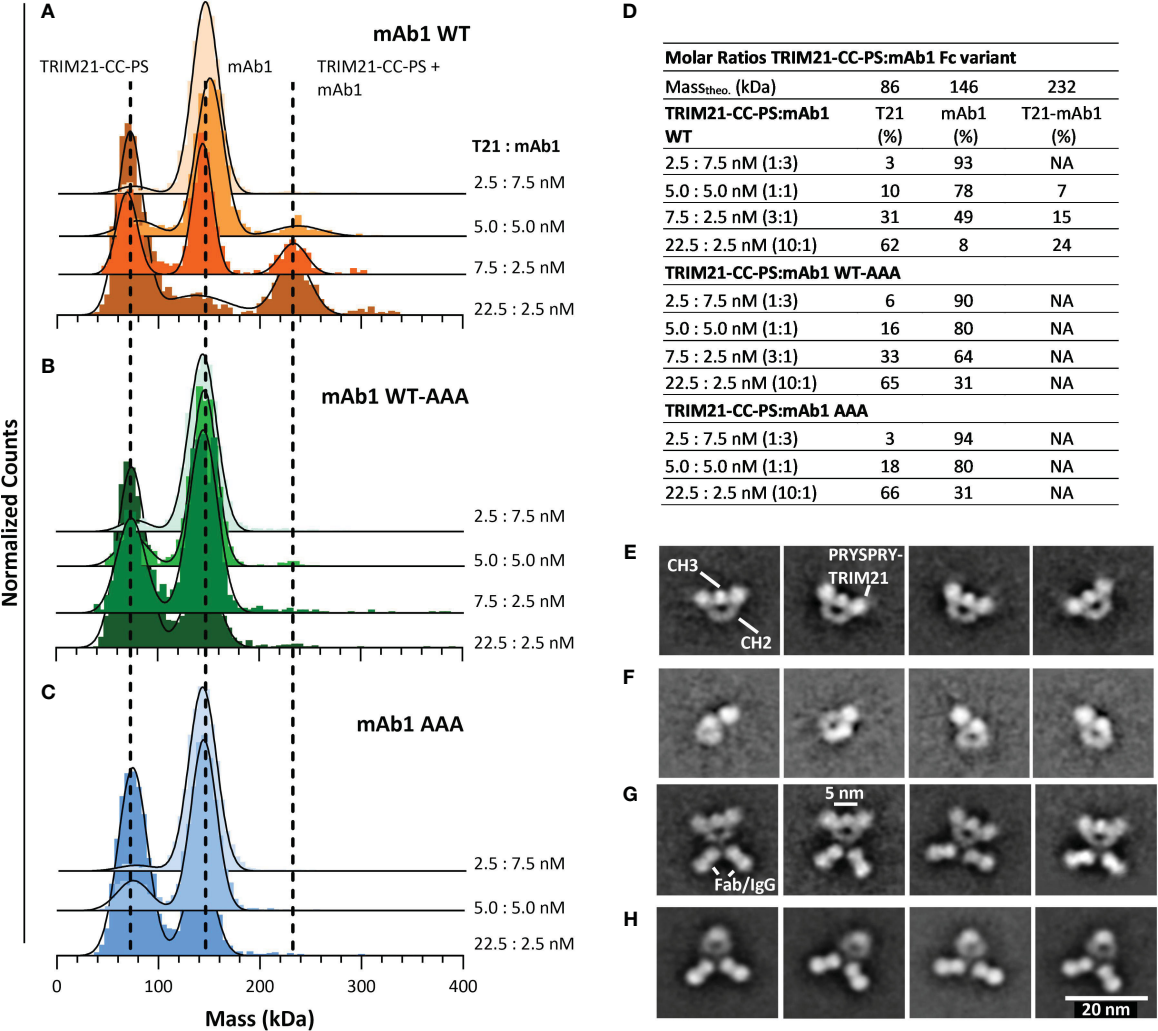
Characterizing the interaction of TRIM21-CC-PS with three different Antibody Fc variants. **(A–D)** Applying MP, dashed lines indicate the main peak of the respective species over all measurements. The applied 3-dimensional Gaussian Fit Distribution is shown in black lines. **(A)** MP of TRIM21-CC-PS with mAb 1 WT, **(B)** MP of TRIM21-CC-PS with mAb 1 WT-AAA **(C)** MP of TRIM21-CC-PS with mAb 1 AAA. Only mAb 1 WT shows binding to TRIM21-CC-PS at low nM concentration in accordance with its low nM binding strength. **(D)** The amount (%) of TRIM21-CC-PS - mAb1 WT complex increases with excess of TRIM21-CC-PS, while TRIM21-CC-PS - mAb1 WT-AAA shows single events of complexed species for the applied concentrations, that could not be fitted robustly. **(E–H)** selected 2D averages of EM data, resolving TRIM21-CC-PS with Fc WT **(E)**, TRIM21-CC-PS with Fc WT-AAA **(F)**, TRIM21-CC-PS with mAb1 WT **(G)** and mAb1 WT alone **(H)**.

To investigate the TRIM21-CC-PS - Antibody Fc binding mode, we applied MP and electron microscopy (**Figure 6**). For the MP measurements we incubated various, increasing TRIM21-CC-PS concentrations with three antibody Fc variants (mAb1 WT, mAb1 WT-AAA and mAb1 AAA) each at different molar ratios.

As shown in **Figures 6A–C**, incubating TRIM21-CC-PS with mAb 1 gave peaks at approximately 80 kDa and 140 kDa, representing uncomplexed TRIM21-CC-PS and mAb1, which is in agreement with their theoretical masses and individual mass measurements (**Figure 5** and SI Info **Supplementary Figure S1**). At approximately 230 kDa appeared a third peak whose molecular weight corresponds to a complex consisting of one TRIM21-CC-PS with one mAb 1 WT (**Figure 6A**). With increasing TRIM21-CC-PS concentration, the equilibrium is shifted to the complexed species until nearly all antibodies are bound. At a 10-fold molar excess of TRIM21-CC-PS (**Figure 6D**), 24% of TRIM21-CC-PS and mAb1 WT are complexed, revealing a 1:1 stoichiometry where one dimer and engages both IgG heavy chains simultaneously (detailed data in SI Info **Supplementary Figure S4**). Detecting the TRIM21-CC-PS - mAb1 WT interaction at low nanomolar concentrations also allows insights into the strong interaction by the dimeric engagement, resulting in an avid binding mode.

The applied mAb1 WT-AAA concentrations showed a minor complexed fraction (approx. 2%), while the AAA variant shows no formed complexes (**Figures 6B, C**). For mAb1 WT-AAA variant, we expect binding, but only to one of the two Fc heavy chains resulting in weak affinity, which cannot be resolved at the concentrations used (similar affinities as for PRYSPRY). The symmetrical AAA mutation abolished TRIM21 binding completely.

To further elaborate the binding mode we analyzed TRIM21-CC-PS with mAb1 WT and the Fc-only variants WT and WT-AAA applying EM (**Figures 6E–H**) and MP (**Supplementary Figure S6**). The results demonstrate that the Fc-only WT-AAA variant binds one PRYSPRY domain, compared to WT (**Figures 6E, F**). The Mab1 WT - TRIM21-CC-PS complex confirms the binding of two PRYSPRY domains to the Fc portion compared to mAb1 WT alone (**Figures 6G, H**). Although the coiled-coil dimerization domain of TRIM21 is not resolved in our 2D class averages, and the analysis of raw images presents challenges due to the size and flexibility of the complexes, the clustering patterns observed in the antibody-TRIM21-CC-PS EM data suggest that a single TRIM21-CC-PS molecule has the capacity to bind to an Fc region through one or both of its PRYSPRY domains. Notably, our data demonstrates that TRIM21 can bind concurrently to two individual Fc regions. This multifaceted binding ability is further corroborated by our AAV binding experiments detailed in section **5.7**. Here, we present evidence that TRIM21 can induce clustering of AAV particles through simultaneous interactions with two different Fc regions of one-armed A20 antibodies.

As depicted in **Figure 5C**, we do not observe a pre-bound/pre-formed positioning of the PRYSPRY domains that would already fit the spatial arrangement of antibodies Fc Part (CH2-CH3). The average distance between the two PRYSPRY domains measures ~10.1 ± 2.1 nm whereas the distance between two Fc-bound PRYSPRY domains is ~5.3 ± 0.5 nm. Therefore, for one TRIM21 to engage both heavy chains, the PRYSPRY domains will have to rearrange. As can be observed in the alphafold model (Uniprot ID P19474), the PRYSPRY domain attaches to its helix with a flexible linker. This linker could allow dimeric engagement by bringing the PRYSPRY domains closer to each other.

### 2.6 Binding kinetic of TRIM21-CC-PS with antibody Fc variants

Here, we examined the interaction between TRIM21-CC-PS and various antibody Fc variants through SPR experiments (assay setup **Figure 1C**), focusing on the avidity binding mode influenced by Fc engineering. Utilizing a low surface density setup to exclude intermolecular interactions, we explored both symmetrical and asymmetrical Fc variants, including YTE, HH, and Y436A, to discern the relationship between antibody affinity and avidity enhancement due to dimeric engagement (previously investigated for affinity binding, **Figure 3**).

**Figure 7:**
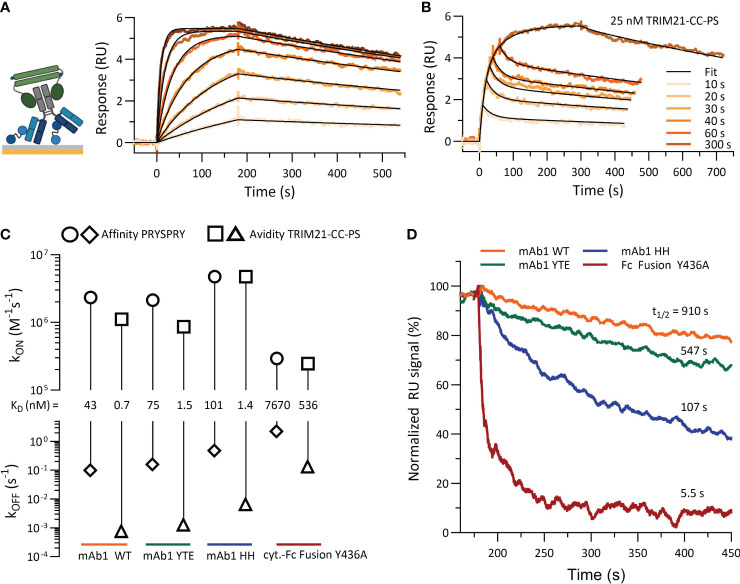
Characterization of TRIM21-CC-PS with Antibody Fc variants. **(A)** shows the sensorgram (SPR) of mAb1 WT (ligand, approx. 8–10 RU) and TRIM21-CC-PS (analyte) where TRIM21-CC-PS was injected in seven different concentration, each for 180 sec as two-fold dilution series with 100 nM as highest concentration. Applied fit model is a simple 1:1 interaction reflecting 100% avid bound, 1:1 antibody - TRIM21-CC-PS species. **(B)** Variation in association time (10 -300 sec) injecting a constant concentration of 25 nM TRIM21-CC-PS to captured mAb1 WT reveals a biphasic binding kinetic, which can be described by applying a two state model providing fast and slow kinetic rates. **(C)** Rate-scale-plot comparing affinity and avidity measurements of Fc variants towards PRYSPRY or TRIM21-CC-PS. **(D)** The altered binding strength from affinity to avidity is mostly off rate driven, which becomes apparent in the overlay of normalized dissociation phases (k_OFF,AVIDITY_) but can also occur as combination of both kinetic rate parameters, namely on and off rate.

**Figure 7** shows the characterization of TRIM21-CC-PS with (antibody) Fc variants. When several antibody concentrations were injected over 180 seconds, a 1:1 fit model describes the data. The dissociation phase is characterized by a significantly reduced dissociation rate (k_OFF,AVIDITY_) in comparison to the affinity-only interactions (k_OFF,AFFINITY_). The sensorgram reveals an avidity only binding mode and a consistent 1:1 stoichiometry (exemplary mAb1 WT, **Figure 7A** and SI Info, **Supplementary Figure S5**).

Variation in the association time for a constant TRIM21-CC-PS concentration (25 nM) with mAb1 WT on the surface (ligand) uncovered a biphasic dissociation pattern, supporting a two-state binding model (**Figure 7B**) (**Equations 4**, **5**). This model illustrates an initial rapid binding and dissociation phase (single bound state, affinity) followed by a slower, more stable avidity-driven complex formation (double bound state, avidity), each with its own association (k_ON_) and dissociation (k_OFF_) rates (**Supplementary Figures S5B, J**). Applying both models results in identical dissociation constants (K_D,AVIDITY_ = 0.7 nM), demonstrating a substantial avidity-enhanced binding strength compared to affinity alone (K_D,AFFINITY_ = 43 nM) (**Figures 7C, D**).

Comparative analysis revealed that avidity enhancements (K_D,AFFINITY_/K_D,AVIDITY_) for Fc variants were significant, with the WT variant showing a 61-fold increase, YTE 50-fold, HH 72-fold, and Y436A 14-fold, when compared to their respective affinities (**Figure 7C**). However, when compared to the WT avidity component, YTE and HH were both about 2-fold less effective, whereas Y436A’s binding strength decreased dramatically by 766-fold. Despite YTE and HH having similar reductions in avidity compared to WT avidity, HH exhibited kinetic rates 5 times faster than YTE. The interaction of asymmetrical antibody Fc variants (WT-AAA, YTE-AAA, HH-AAA) with TRIM21-CC-PS showed only the affinity binding mode (SI Info, **Supplementary Figure S5**).

Complementary MP analysis (SI Info, **Supplementary Figure S6** and **Supplementary Table S2**) corroborated our SPR findings, confirming a 1:1 stoichiometry for all symmetrical constructs and showing a trend where weaker bindings resulted in fewer avid complexes as the identical the concentration of antibody-TRIM21-CC-PS was used. Additional MP experiments with other antibody Fc WT constructs, such as mAb2 WT, Briakinumab, and Ustekinumab, revealed similar complexation levels to mAb1 WT, indicating that the variable domains of these antibodies do not affect their interaction with TRIM21-CC-PS. This further validates our SPR-derived conclusions.

### 2.7 TRIM21 within the context of viral neutralization

**Figure 8:**
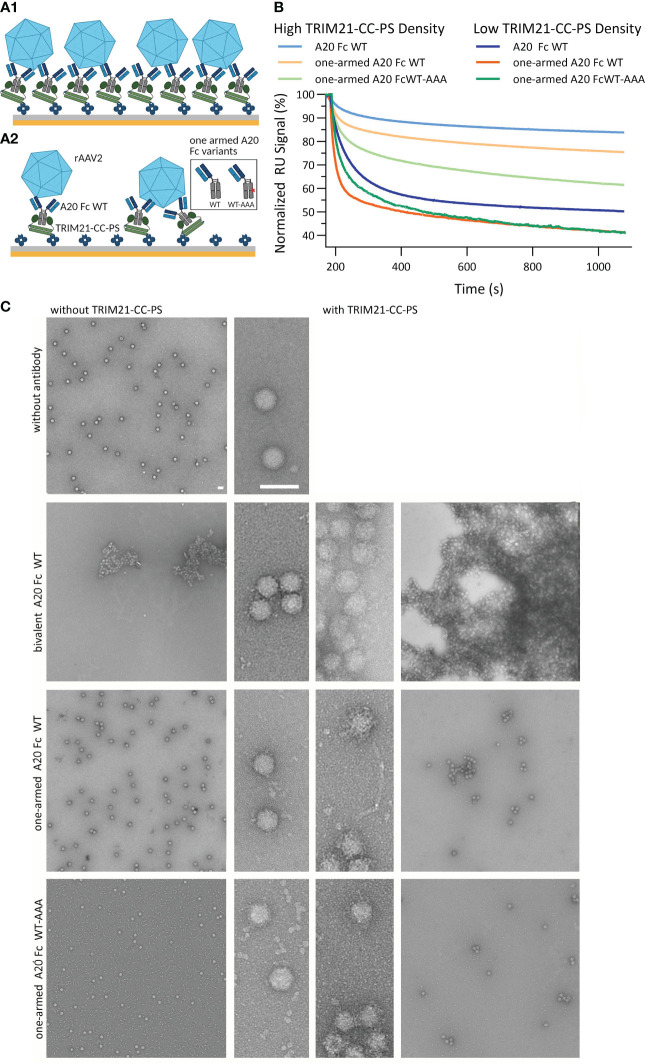
TRIM21-CC-PS-Antibody-AAV2 Characterization. SPR Assay data is shown in **(A, B)**. **(A1, A2)** Schematic SPR assay configuration to analyze the affinity to avidity interplay of TRIM21-CC-PS, anti-capsid antibody variants A20 and rAAVv-2. Biotinylated TRIM21-CC-PS is captured via monovalent streptavidin achieving a captured level of 190 RU (**A1**, high density) and 35 RU (**A2**, low density). Subsequent, anti-AAV2 capsid antibody variants (bivalent A20 Fc WT, one-armed A20 Fc WT and Fc WT-AAA) are injected to saturate the TRIM21-CC-PS surface, followed by the injection of rAAV-2. **(B)** Overlay of the normalized dissociation phases (Start of Dissociation: 100%) after the injection of 3.32 nM rAAVv-2 over low and high TRIM21-CC-PS-A20 densities. At higher antibody densities, more avid complexation occurs and a higher degree of rAAVv-2 surface decoration is possible. This allows less complex to dissociate over time to due to simultaneous engagement of both, TRIM21-CC-PS and AAV2, mediated via the A20 antibody variants. **(C)** Electron microscopy images of rAAVv-2 interactions with antibodies alone (left column) and TRIM21 additionally (right column). The scale bars represent 50 nm.

Determining whether TRIM21 compromises the efficacy of AAV-based gene therapies is critical, yet its role has not been established. To explore this potential involvement, our study focuses on the interactions between TRIM21 and recombinant adeno-associated virus vector (rAAVv) particles. We employed SPR to simulate the binding of TRIM21 to antibody-coated rAAVv particles (**Figure 8A**). Additionally, we have used electron microscopy (EM) to visually document these interactions, with findings depicted in **Figure 8C**. Our setup simulates the scenario after endosomal escape of antibody decorated AAV particles and their encounter with cytosolic TRIM21, proposing that TRIM21’s interaction with antibody-bound virus particles triggers ubiquitination through intermolecular RING dimerization, as suggested previously (20, 53). Our assay involved TRIM21-CC-PS, anti-AAV2 capsid antibody A20 variants (including a bivalent A20 with WT Fc and IgG variants with a single Fab arm against rAAVv-2, featuring either WT Fc or WT-AAA asymmetrical Fc), and rAAV serotype 2 (rAAV2), as depicted in (**Figure 8A**). Our research reveals complex interactions between TRIM21-CC-PS, A20 antibody variants, and rAAVv-2, showing that binding dynamics are more intricate than simple models suggest (**Figure 8B**). These interactions involve both affinity and avidity effects, with TRIM21-CC-PS binding to the antibodies Fc region and the Fab arm(s) binding to the rAAVv-2 capsid, contributing both to overall avidity.

All three anti-AAV2 antibody variants show affinities for the capsid in the same range (K_D,AFFINITY_ 75 - 140 nM), whereas the bivalent A20 WT also demonstrated avid binding K_D,AVIDITY_ = 5 nM SI Info, (**Supplementary Figure S7**). The full IgG A20 Fc WT shows strong bivalent engagement with TRIM21-CC-PS (K_D,AVIDITY_ = 0.7 nM) unlike the asymmetrical AAA-WT variant, which lacks this avidity SI Info (**Supplementary Figure S8**).

The AAV2 viral capsid harbors n = 60 epitopes for A20, assuming intra- and intermolecular bivalent binding is possible, depending on antibody variant and concentration (61). By varying the A20 antibody levels (8-fold different capture level, **Figure 8B**) and analyzing EM data (**Figure 8C**), we observed that higher antibody densities on the virus surface enhance avidity. The bivalent A20 Fc WT variant promotes increased avidity, surpassing the monovalent A20 Fc WT and WT-AAA variants in binding efficiency, leading to faster dissociation.

Analysis of the dissociation phases indicate that all antibody variants demonstrate avidity, with slower and faster dissociating species suggesting complex formation between rAAVv-2 and antibodies. On a biosensor surface, ligands are randomly distributed by statistics. As such, two adjacent, single arm A20 constructs could still interact with one rAAVv, allowing avidity to occur. However, lower antibody densities result in a greater proportion of fast dissociating species, indicating a decrease in avidity (**Figure 8B**, low surface density).

EM images (**Figure 8C**) reveal that A20 antibodies as well as TRIM21-CC-PS facilitate AAV particle aggregation, owing to their ability to bind two separate molecules. While A20 causes severe aggregation, to the point that an additional effect of TRIM21 cannot be measured, the one-armed antibodies lose their ability to cause aggregation, allowing us to separate the effect of TRIM21 (**Figure 8C**, bottom two rows). Indeed, TRIM21 causes aggregation, albeit to a lesser extent. This confirms our SPR observation that reducing bivalent engagement modifies but does not negate avidity. Our findings emphasize the crucial role of the antibody as an immune regulator in mediating both, target binding (AAV) and effector functions via TRIM21 engagement achieving avidity, demonstrating the multifaceted nature of these interactions.

## 3 Discussion

This study systematically explores the interaction of TRIM21 with antibody Fc variants through SPR, MP, and EM to elucidate antibody binding to TRIM21 and investigate a TRIM21-mediated AAV neutralization mechanism. We meticulously optimized our SPR assays for each scientific hypothesis, facilitating low-density studies ideal for distinguishing between affinity and avidity. This approach yielded significant insights into the dynamics and mechanisms of TRIM21 interaction with human IgG Fc variants including a newly proposed binding mechanism.

TRIM21 is a highly conserved mammalian Fc receptor that is structurally and mechanistically distinct from previously identified Fc receptors (22). Our analysis confirmed the PRYSPRY–mAb1 WT interaction affinity at 43nM, aligning closely with prior ITC data (37 nM) (24) and differing from previous SPR results (212 nM) (62). This discrepancy originates from our refined assay conditions, which resulted in a 5-fold increase in binding rate (k_ON_) without altering the dissociation rate (k_OFF_). Our methodology, mirroring ITC gold-standard accuracy, demonstrates the benefits of optimized assay setups for enhanced target interaction analysis, thereby addressing the discrepancies observed in prior SPR experiments. Additionally, our findings on the mAb1 WT-AAA Fc variant versus symmetrical Fc WT confirmed a 2:1 stoichiometry and identical affinities of two TRIM21 PRYSPRY domains for one IgG Fc homodimer (K_D,AFFINITY,WT-AAA_ = 40 nM vs K_D,AFFINITY,WT_ = 43 nM), supporting a non-cooperative binding model (24). This aligns with the symmetric TRIM21-IgG interaction in the previously reported crystal structures (24).

Despite TRIM21 and FcRn sharing the Fc CH2-CH3 binding site, the HNHY motif, mutations in the Fc region affect their interactions differently. We engineered mutations within this interface (YTE, HH, Y436A, AAA), previously identified to impact FcRn binding, to assess their effect on TRIM21 (33, 34, 38–41, 63). Our findings reveal a ranking of affinities for PRYSPRY, from strong to weak: WT, YTE, HH and Y436A. Notably, the Y436A mutation significantly weakens the binding to both FcRn (41) and TRIM21, with a 180-fold decrease in TRIM21 binding affinity, a previously unexplored aspect in the context of TRIM21 interaction. Through SPR analysis, we confirmed an avid equilibrium dissociation constant (K_D,AVDIDITY_) of 0.7 nM for mAb1 Fc WT (vs. 0.6 nM as determined by fluorescence anisotropy) and confirmed a 1:1 stoichiometry (9). In addition, we uncovered kinetic parameters alongside with an avidity enhancement (K_D,AFFINITY_/K_D,AVIDITY_) for the Fc variants of 14- to 72-fold. This suggests a substantial affinity to avidity transition due to bivalent engagement, particularly notable with the Y436A mutation, which demonstrates affinity in the µM range, while avidity is nM.

Interestingly, while the YTE and HH variants weaken TRIM21 binding compared to WT, they enhance FcRn binding (10-fold for YTE, HH is in similar range); in a pH-dependent manner, highlighting a divergent influence of these mutations on the two receptors (33, 34, 38). The AAA mutation, which eliminates FcRn binding (39, 40), also abolishes TRIM21 interaction. Remarkably, we found no Fab contribution that adds an additional complexity to TRIM21 as it does for FcRn (42, 43). A possible hypothesis to explain this phenomenon could be the pH independency known for TRIM21 (22). The divergence of TRIM21 and FcRn suggests potential implications for efficient antibody recycling and ADIN. A potential intracellular transition between FcRn and TRIM21 has to be explored. Our study emphasizes the importance of considering both affinity and avidity in TRIM21 interactions and challenges the direct extrapolation from one to the other, underlining the complexity of antibody engineering with respect to receptor binding.

Our configuration of unbound TRIM21 revealed that the C-terminal PRYSPRY domains within a TRIM21 dimer, where dimerization is mediated via the coiled coil domains, are positioned at opposite ends, away from each other (64). This finding is in contrast to previous studies on TRIM family proteins, such as TRIM25. It has been suggested, that unbound C-terminal domains, responsible for substrate binding, are centrally located within an elongated structure characterized by a coiled-coil domain (65, 66). A possible explanation for the changed TRIM21 configuration in the unbound state is the truncation of TRIM21 (TRIM21-CC-PS). Arguing against this is the fact, that the length of the coiled coil domain exceeds the distance measured between the two PRYSPRY domains by at least 3 nm. Also, the truncated sequence still maintains a stretch of 30 amino acids beyond the coiled-coil domain, suggesting a natural configuration at the observed binding location. Our analysis demonstrates that the PRYSPRY domains are spaced approximately 6–12 nm apart, diverging from the ~5 nm separation when bound to Fc, which challenges the notion of a pre-bound state compatible with the Fc structure and spatial arrangement (**Figures 5**, **6**).

**Figure 9:**
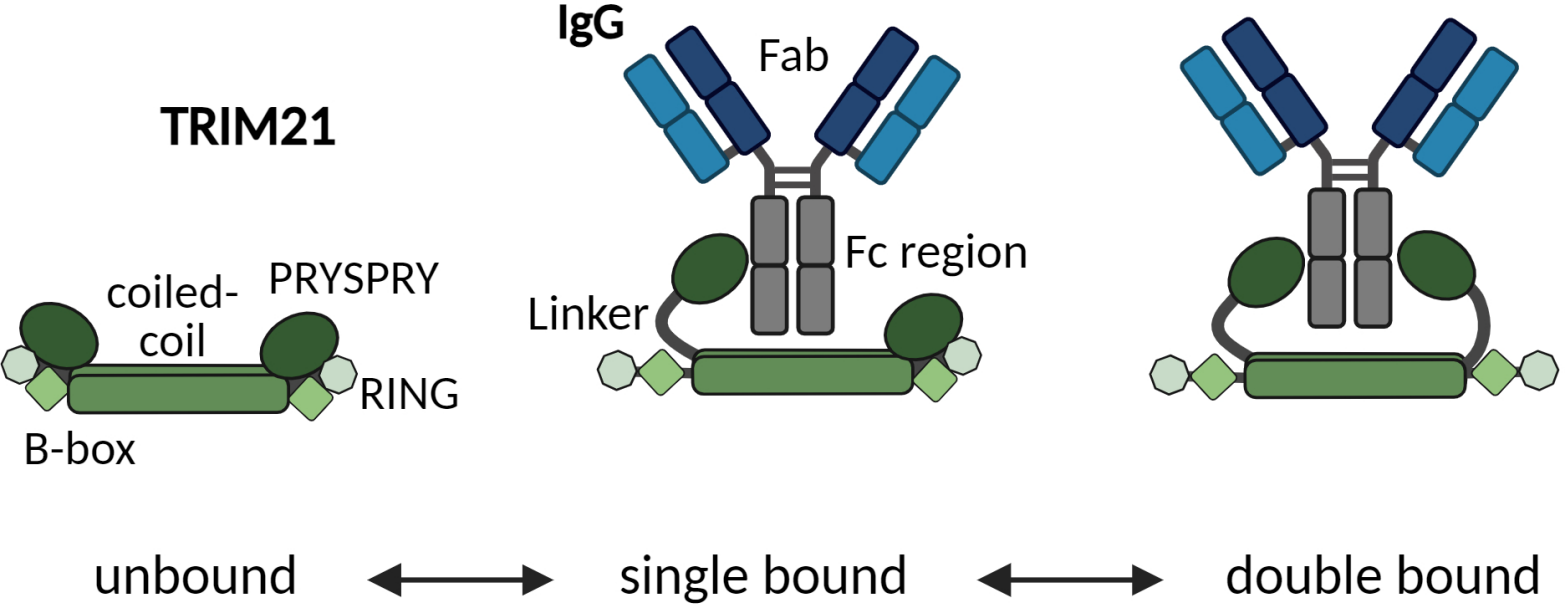
Schematic model suggesting how one TRIM21 dimer engages both sites of the Fc region in a two-step process. Upon Fc binding, the PRYSPRY detaches from the coiled-coil domain. The linker domain allows enough freedom of movement to allow engagement of the second PRYSPRY domain. Only after initial binding bivalent engagement of both Fc heavy chains is possible.

Zeng and colleagues (53) have proposed a TRIM21-IgG Fc-complex structural framework based on Small-angle X-ray scattering (SAXS) data, positioning the bound PRYSPRY domains centrally, which is in agreement with our findings. This configuration also accounts for avidity due to bivalent Fc engagement, observed in our SPR (**Figure 7C**) and MP data (**Figure 6A**), as well as the positioning of the bound PRYSPRY domain indicated by our negative staining techniques (**Figures 6E–H**). Beyond previous findings, our detailed examination underpins a novel conceptual framework for TRIM21 structural dynamics in both its bound and unbound states (**Figure 9**). In its unbound state, the dimer’s PRYSPRY domains converge at the coiled coil’s apex, a feature discernible through negative staining (**Figure 5C**). Binding to an Fc site results in the detachment of PRYSPRY from the coil, enhancing mobility due to its flexible linker, and thereby facilitating the engagement of the second site in a spatially favorable configuration. Our SPR data confirms, that binding occurs in a two-step process, initially rapid, followed by a slower event that forms the final TRIM21-IgG complex (**Figure 7B**). The slower phase occurs over a prolonged duration to accommodate the Fc geometry (**Supplementary Figure S5J**, k_ON2_ = 0.02s^-1^). The transition markedly increases the complex flexibility, rendering the coiled-coil domain indistinct in negative stain averages of the TRIM21-CC-PS - Fc assembly.

Integrating the structural insights from PRYSPRY domains in complex with Fc (PDB-ID: 2IWG) (24) with the predictive model of TRIM21 (Uniprot ID: P19474) and its dimeric form according to AlphaFold2 (for details see materials and methods) yields diverse models (67). While a minority retains the dimeric form of TRIM21 engaging a single Fc site (**Supplementary Figure S10A1**), most predictions indicate a separation of PRYSPRY domains from the coiled-coil (**Supplementary Figures S10A2, B**) agreeing with our model (**Figure 9**).

Combining our findings, we propose a tentative model in which the PRYSPRY engagement with the Fc domain may influence its interactions with other domains of TRIM21. This observation leads us to speculate that within a TRIM21 dimer, the PRYSPRY domain might have an additional, yet uncharacterized, role in modulating the interactions of the B-Box and the RING domains. We suggest that the binding of the PRYSPRY domain to the Fc domain could potentially release these intra-domain interactions, thereby affecting RING activation.

Lastly, our study delved into the TRIM21 antiviral mechanism, particularly its role in E3 ligase activation and neutralization of adeno-associated virus (AAV) using AAV as a model. We investigated how the anti-AAV2 capsid antibody A20 interacts with TRIM21 and rAAVv-2, noting A20 unique ability to neutralize AAV2 and AAV3 by targeting a specific conformational epitope (68, 69). Despite the lack of knowledge regarding the precise neutralization mode, subsequent to initial heparan sulfate proteoglycan (HSPG) primary receptor attachment, our data suggests A20 impedes viral entry by promoting large aggregate formation (**Figure 8**), potentially blocking endocytosis-mediated cell entry (61, 69, 70). We highlight the distinction between neutralizing and non-neutralizing antibodies in human blood, with the latter capable of cytosolic entry, as antibody-AAV complexes, post-endosomal escape (71, 72). However, the role of non-entry blocking antibodies in TRIM21-mediated AAV neutralization remains unexplored due to their unavailability for testing. Our findings indicate antibody clustering on AAV is feasible, aiding in the activation of TRIM21’s E3 ligase via RING dimerization, a critical step in neutralization (9, 20, 53). Comparing AAV with AdV, we noted the smaller size of AAV and fewer antibody binding sites could significantly influence its neutralization process. Unlike A20, which forms large aggregates, the anti-AdV5 antibody does not aggregate virions or hinder their attachment nor trafficking (3, 4, 44, 73, 74).

Our elucidated TRIM21 dimeric nature and its interaction with Fc-engineered antibodies revealed a new mechanism for bivalent engagement and demonstrated the impact of Fc mutations on both affinity and avidity. This knowledge is pivotal for advancing antibody engineering and understanding TRIM21 function in viral defense.

## 4 Materials and methods

### 4.1 Recombinant TRIM21 variants

Recombinant human TRIM21 proteins, including the PRYSPRY domain variant and the PRYSPRY-coiled-coil (TRIM21-CC-PS) variant, were produced and purified by Proteros Biostructures GmbH (Planegg, Germany). The production process involved the transformation of Escherichia coli strain BL21 DE3, the overexpression of TRIM21 under standard conditions (18°C, 1mM IPTG, 16h) and the purification by Ni-NTA resin (Qiagen, Hilden, Germany) and Superdex S-200 26/60 gel filtration (GE Healthcare, IL, US). Each plasmid used for the transformation encoded either the TRIM21 PRYSPRY domain or the TRIM21-CC-PS variant. Both variants were C-terminally fused to a His-Avi-Tag via a (4GS)_1_ linker. The gene used for this process is identified by NCBI Gene ID 6737, which corresponds to UniProtKB identifier P19474–1 (Isoform 1). The amino acid sequence for the TRIM21 PRYSPRY variant spans from amino acid 277 to 475, while the sequence for the TRIM21-CC-PS variant spans from amino acid 128 to 475. To verify the protein integrity SDS PAGE, SEC Analysis, Mass Photometry and Mass Spectrometry for mass identification were performed.

### 4.2 Antibody constructs/Fc variants

Recombinant human IgG1 Fc wildtype, Fc variants, Fc-only variants (core hinge-CH2-Ch3) with specific mutations (Fc WT, Fc WT-AAA, mAb1 WT, mAb1 WT-AAA, mAb1 AAA, mAb1 YTE, mAb1 YTE-AAA, mAb1 HH, mAb1 HH-AAA, mAb2 WT, mAb2 WT sym., anti-AAV2 capsid antibody A20 WT, A20 one arm WT, A20 one arm WT-AAA), and cytokine-Fc Fusion constructs (cytokine-Fc Fusion WT, cytokine-Fc Fusion WT-AAA, cytokine-Fc Fusion Y436A) as well as Ustekinumab and Briakinumab were produced and purified internally at Roche (Penzberg, Germany). Mutations (Kabat numbering scheme) are the following: WT: wildtype; AAA: H310A, H433A, Y436A; YTE: M252Y, S254T, T256E; HH: T307H, N434H and Y436A. The production process involved the transient transfection of HEK Expi293F cells, sourced from Thermo Fisher Scientific (MS, US), with plasmids encoding both the light and heavy chains. Asymmetrical variants were generated using the knob-into-hole technology (75). This technology refers to refers to mutations Y349C, T366S, L368A and Y407V (Hole) and S354C and T366W (Knob) both in the CH3-CH3 interface to promote heteromultimer formation (76, 77).

### 4.3 Virus supply

Replication-deficient, recombinant Adeno-associated virus vector particles serotype 2 (rAAVv-2) without carrying any transgene (empty rAAVv-2) were supplied by Virovek (CA, US), aliquoted up on arrival and frozen at -80°C until required. The electron microscopy experiments were performed using genome-filled rAAV2 vectors, supplied by Sirion Biotech (Germany).

### 4.4 SEC-MALS

The molecular weight of the sample was determined using size-exclusion chromatography combined with multi-angle light scattering (SEC-MALS). The SEC-MALS analysis was performed using a high-performance liquid chromatography (HPLC) instrument (Superdex200 Increase 10/300 GL, GE Healthcare). The HPLC instrument was coupled with a Wyatt Heleos II 18-angle light-scattering instrument (297-TS, Wyatt Technology) and a Wyatt Optilab rEX (766-rEX, Wyatt Technology) online refractive index detector. The sample was prepared at a concentration of 1 mg/mL, and 50 µg of the sample was injected into the system. The running buffer used was phosphate-buffered saline (PBS) at pH 7.4. The system was operated at a flow rate of 0.5 mL/min. To ensure the accuracy and reliability of the system, a system suitability test was performed by injecting bovine serum albumin (BSA) prior to the sample analysis. The results from the BSA injection were used to validate the performance of the system and to confirm that it was operating within the acceptable parameters for accurate molecular weight determination. The Zimm model has been used as a default data process procedure in ASTRA 8.2 (Wyatt Technology).

### 4.5 SPR experiments

#### 4.5.1 General assay setup

The Biacore T200 system (GE Healthcare, Uppsala, Sweden) was used for all surface plasmon resonance (SPR) experiments. The C1 sensor chips (carboxymethylated matrix-free surface), amine coupling kit (containing 1-ethyl-3-(3-dimethylaminopropyl) carbodiimide hydrochloride (EDC), N-hydroxysuccinimide (NHS), and ethanolamine-HCl), HBS-N (10 mM HEPES, 150 mM NaCl, pH 7.4), HBS-EP+ running buffer (10 mM HEPES, 150 mM NaCl, 3 mM EDTA, 0.05% (v/v) surfactant P20, pH 7.4), and regeneration solutions were also obtained from Cytiva.

The ligand was immobilized on the C1 sensor chip using standard amine coupling chemistry. Briefly, the sensor surface was conditioned with two one minute injections of conditioning solution (0.1 M glycine-NaOH, 0.3% (v/v) Triton X-100, pH 12) followed by “Extra wash” and then run “Prime” using HBS-N running buffer. The Chip was then activated by injecting a mixture of 0.4 M EDC and 0.1 M NHS at a flow rate of 10 µL/min for 7 minutes. The ligand was then injected at a concentration of 20 µg/mL in 10 mM sodium acetate buffer (pH 4.5) at a flow rate of 10 µL/min for 3 minutes unless stated otherwise. Unreacted groups were blocked by injecting 1 M ethanolamine-HCl (pH 8.5) at a flow rate of 10 µL/min for 7 minutes.

Next, a molecule of interest, diluted accordingly in HBS-EP+ running buffer was captured via the amine coupled ligand to a desired Response unit (RU) level at a flow rate of 5 µL/min.

Binding kinetics were measured at 25°C by injecting various concentrations of the analyte over the captured molecule of interest at a flow rate of 50 µL/min unless stated otherwise. Each injection was followed by a dissociation phase. The sensor surface was regenerated between each cycle by injecting 10 mM glycine-HCl (pH 2 or 3, see below) at a flow rate of 30 µL/min.

The sensorgrams were analyzed using the Biacore T200 evaluation software (v3.1) and GraphPad Prism (v8.4.2). The data were fitted to a 1:1 binding model to determine the association rate constant (k_ON_), dissociation rate constant (k_OFF_), and equilibrium dissociation constant (K_D_). The K_D_ was calculated as k_OFF_/k_ON_. For analyzing complex binding kinetics the data were fitted by applying the Two State Reaction Model obtaining a K_D_ value explained in more detail in the following section. The results are presented as mean ± standard deviation.

To ensure the reliability of the data, a blank run (buffer only) was performed before each experiment to check the baseline stability.

#### 4.5.2 TRIM21 PRYSPRY injections to captured antibody (Fc) Variants, immobilized Fc only variants and captured cytokine-Fc fusion constructs

To investigate the interactions of TRIM21 PRYSPRY with various protein constructs, we utilized a consistent methodology across different experiments, applying standard amine coupling chemistry for immobilization on a C1 sensor chip. The experiments focused on three targets: antibody (Fc) variants, Fc only variants, and cytokine-Fc Fusion constructs.

For the antibody (Fc) variants and Fc only variants, we aimed for initial immobilization to achieve response unit (RU) signals of approximately 60 for antibody variants and between 60 and 80 for Fc only variants, using the CaptureSelect™ Human Fab-kappa Kinetics Biotin Conjugate ligand (7103302100, Thermo Fisher Scientific) for the former and direct immobilization for the latter. The cytokine-Fc Fusion constructs involved capturing using an in-house anti-PGLALA F(ab’)2 fragment (55) with a target RU signal between 35 and 40.

Subsequent steps involved injecting five different concentrations of TRIM21 PRYSPRY, prepared in a two-fold dilution series, across all experiments. Each concentration underwent an association phase of 60 seconds and a dissociation phase of 180 seconds. The integrity of the sensor surface for subsequent cycles was maintained through a regeneration process involving twice injections of 10 mM glycine-HCl (pH adjusted according to the specific experiment - pH 2.0 for antibody variants and cytokine-Fc fusions, pH 3.0 for Fc only variants) for 60 seconds each, followed by a 120-second injection of running buffer (HBS-EP+) at a flow rate of 30 µL/min. This ensured the complete removal of any bound molecules from the previous cycle and restoration of the sensor surface to its initial state, facilitating accurate and repeatable measurements across the experiments.

#### 4.5.3 Antibody (Fc) variants/Fc only variants/cytokine-Fc fusion constructs injections to captured TRIM21 PRYSPRY

In the initial step, a monovalent Streptavidin (mono-SA, in house, Roche) was immobilized on a C1 sensor chip via standard amine coupling chemistry. The ligand was introduced at a concentration of 10 µg/mL in 10 mM sodium acetate buffer (pH 4.5) at a flow rate of 10 µL/min for 180 seconds. Subsequently, biotinylated TRIM21 PRYSPRY in running buffer (HBS-EP+) was captured via mono-SA, aiming for a response unit (RU) signal of 3 to prevent any inter-molecular interactions interlinking two TRIM21 PRYSPRY by one antibody. Following this, five different concentrations of TRIM21 PRYSPRY, as specified in the sensorgrams, were prepared in a two-fold dilution series and introduced over the sensor chip. Each concentration underwent a 60-second association phase, followed by a 180-second dissociation phase to monitor the rate of complex decay. To restore the sensor surface for subsequent cycles, a regeneration step was performed after each cycle. This involved the injection of 10 mM glycine-HCl (pH 3.0) twice, each for 15 seconds, followed by a 30-second injection of running buffer (HBS-EP+) at a flow rate of 20 µL/min. This process ensured the complete removal of any bound molecules from the previous cycle and the restoration of the sensor surface to its initial state.

#### 4.5.4 TRIM21-CC-ps injections to immobilized antibody (Fc) variants or cytokine-Fc fusion constructs

First, CaptureSelect™ Human Fab-kappa Kinetics Biotin Conjugate ligand (7103302100, Thermo Fisher Scientific) to capture Antibody (Fc) Variants or anti-PGLALA F(ab’)2 fragment (in-house, Roche) to capture cytokine-Fc Fusion constructs was immobilized. The ligands were introduced at a concentration of 5 µg/mL in 10 mM sodium acetate buffer (pH 4.5) at a flow rate of 5 µL/min for 120 seconds. Second, TRIM21-CC-PS in running buffer (HBS-EP+) was captured via the ligand, aiming for a response unit (RU) signal of 8. Following this, several different concentrations of Trim2-CC, as specified in the sensorgrams, were prepared in a two-fold dilution series and introduced over the sensor chip. Each concentration was injected for 180-second association phase, followed by a 600-second dissociation phase. To restore the sensor surface for subsequent cycles, a regeneration step was performed after each cycle. This involved the injection of 10 mM glycine-HCl (pH 2.0) twice, each for 60 seconds, followed by a 120-second injection of running buffer (HBS-EP+) at a flow rate of 30 µL/min.

#### 4.5.5 Mimicking an TRIM21-CC-PS – antibody decorated viral complex

The formation of an immune complex on a sensor chip, consisting of TRIM21-CC-PS, an antibody, and AAV2-wt as a viral model, was achieved using surface plasmon resonance (SPR) technology on a Biacore system. Initially, monovalent Streptavidin (mono-SA, in house, Roche) was immobilized on a C1 sensor chip (Flow cells 1–4) until saturation was reached. This was achieved by injecting mono-SA at a concentration of 10 µg/mL in 10 mM sodium acetate buffer (pH 4.5) at a flow rate of 5 µL/min for 120 seconds using standard amine coupling chemistry. The running buffer during this step was HBSN. Following a buffer change to HBS-EP, biotinylated TRIM21-CC-PS was captured on flow cell 2 to approximately 35 RU and on flow cell 3 to approximately 190 RU to generate different ligand densities. Flow cell 1 served as a reference surface. Subsequently, different anti-AAV2 capsid antibody constructs with TRIM21 relevant Fc mutations were injected at a concentration of 5 µg/mL at a flow rate of 20 µL/min for 180 seconds until TRIM21-CC-PS was saturated. Following this, three different concentrations of AAV2-wt-empty, as specified in the sensorgrams, were prepared in a three-fold dilution series and introduced over the sensor chip. Each concentration underwent a 180-second association phase, followed by a 900-second dissociation phase at a flow rate of 10 µL/min. To restore the sensor surface for subsequent cycles, a regeneration step was performed after each cycle. This involved the injection of 10 mM glycine-HCl (pH 3.0) for 20 seconds, followed by a 30-second injection of running buffer (HBS-EP+) at a flow rate of 20 µL/min.

#### 4.5.6 1:1 Binding model

In a simple 1:1 interaction, an analyte (A) binds to a ligand (B) to form a complex (AB). The equilibrium dissociation constant (K_D,AFFINITY_) was calculated using the equation:


(1) $$K_{D,AFFINITY}=\frac{k_{OFF}}{k_{ON}}$$

R_max_ (maximum response) is a parameter that represents the maximum binding capacity of the ligand immobilized on the sensor chip surface. It is determined by the amount of ligand immobilized and the stoichiometry of the interaction. R_max_ is measured in response units (RU). This equation assumes that all the immobilized ligand is active and capable of binding to the analyte. In practice, not all the immobilized ligand may be active, and the observed R_max_ may be less than the calculated Rmax.


(2) $$R_{max}\left(RU\right)=\frac{R_{ligand}\;*\;MW_{analyte}\;*\;Valency_{ligand}}{MW_{ligand}}$$

MW_analyte_ is the molecular weight of the analyte. MW_ligand_ is the molecular weight of the ligand. R_ligand_ is the immobilization level of the ligand, measured in RU. Valency_ligand_ is the number of analyte molecules that bind to each ligand molecule.


(3) $$R_{max,ratio}\left(\%\right)=\frac{R_{max,experimentell}}{R_{max,theory}}$$

The R_max,ratio_, calculated as provides a measure of the proportion of active ligand and thereby the Stoichiometry.

#### 4.5.7 Complex binding kinetics – two state reaction

The rationale behind the Two State Model lies in its ability to account for the additional kinetic steps that occur during the binding process. In contrast to a 1:1 binding model, in some interactions, the formation of the initial complex induces a conformational change, leading to a different, usually more stable, complex (AB*). This can be represented as follows:


(4) $$A+B↔AB↔AB^{*}$$

In this model, the analyte first associates with the ligand with an association rate constant (k_ON1_), forming an initial complex (AB). This complex then undergoes a conformational change with a rate constant (k_ON2_) to form the final complex (AB*). Each of these steps is reversible, with the initial complex dissociating back to the free analyte and ligand with a rate constant (k_OFF1_), and the final complex reverting back to the initial complex with a rate constant (k_OFF2_).

The apparent or avidity equilibrium dissociation constant (K_D_) reflects the overall stability of the final AB* complex and is calculated using the formula:


(5) $$K_{D,AVIDITY}=\frac{k_{OFF1}}{k_{ON1}}\;*\;\left(\frac{k_{OFF2}}{\left(k_{OFF2}+k_{ON2}\right)}\right)$$

### 4.6 Mass photometry technology

The mass photometry experiments were conducted using the OneMP system, TwoMP system and OneMP-MassFluidix HC system to analyze low-affinity interactions (Refeyn Ltd., Oxford, UK) at room temperature. The sample buffer used for all experiments was 1x PBS, pH 7.4 (Roche Diagnostics GmbH). The sample carrier slides (Refeyn Ltd., Oxford, UK) were prepared by cleaning them in an isopropanol ultrasonic bath for 10 minutes, followed by consecutive rinsing with H_2_O. The slides were then dried under a stream of clean nitrogen. A fourteen well, each well 3 mm in diameter, sample well gasket (Refeyn Ltd., Oxford, UK) providing four measurement spots, each capable of holding a volume of 20µL sample solution, was placed onto the carrier slide. The molecules of interest, TRIM21 PRYSPRY or TRIM21-CC-PS with antibody Fc variants, were prepared in the sample buffer at various concentrations, specifically four times higher than in the final droplet (for final concentrations and ratios, see histograms). The samples were thoroughly mixed and incubated at room temperature for 10 minutes to allow for complex formation. Approximately 5 µL of the sample was placed on a clean glass coverslip, containing 15µL of PBS, and loaded into the mass photometry system. The system was set to acquire data using the AquireMP 2.5 software (Refeyn Ltd.) for a period of 1 minute at a 1 kHz frame rate. The mass photometry data were analyzed using DiscoverMP software 2024 R1 (Refeyn Ltd.) and GraphPad Prism (Version 8.4.2, GraphPad Software, Inc., CA, USA). A 3-dimensional Gaussian distribution model was applied for the analysis. The ratio (in %) for each detected species, whether a single molecule or an interacting pair of molecules, was calculated by dividing the counts of events within a specific Gaussian distribution by the total number of detected events. The acquisition parameters were set according to the manufacturer’s instructions and optimized for the specific molecules of interest using the following parameters: number of averaged frames: 5, threshold 1: 1.5, and threshold 2: 0.25. The mass of the individual molecules and their complexes were determined from the intensity of the light scattered by the molecules. Results were reported as normalized counts, calculated by dividing events in each bin by the total number of events. To ensure the reliability of the data, a control experiment was performed with each molecule alone to confirm their individual masses (5 nM concentration in the final droplet). A mass calibration curve was generated by analyzing three different proteins with known molecular masses of approximately 66 kDa, 145.5 kDa, and 194 kDa, respectively (Roche Diagnostics GmbH). The calibration data was collected from the 2.9 μm × 10.8 μm instrument field of view for 100 seconds.

### 4.7 Electron microscopy experiments

Samples were diluted in D-PBS (Gibco Life Technologies) until a suitable concentration was reached for negative staining EM. Interaction mixtures (**Figure 6**) were mixed and incubated at RT for 1h prior to dilution and imaging. Recombinant AAVv-2-antibody interactions (**Figure 8**) were incubated for 30 minutes except for bivalent A20 Fc WT, which caused full precipitation at 30 minutes and was incubated for 5 minutes only. Images that additionally contain TRIM21-CC-PS were incubated for 12 minutes with antibody variants, followed by 18 minutes of incubation with TRIM21-CC-PS. Since bivalent A20 Fc WT alone was sufficient to fully precipitate rAAVv-2s, it was not possible to observe an additional effect of TRIM21-CC-PS. Electron microscopy grids (T600H-Cu 698 l/inch Hex. mesh Thin Bar; EMS) were coated with a home-made ~2nm carbon film by floating the carbon on H2O and letting the water level drop till the carbon covered the grids. After at least 2 days of drying the grids were used. Four ul of sample was incubated on a glow-discharged carbon coated grid for 30 seconds, followed by two steps of washing with H2O, a step of washing with UAc (2%) solution and incubation with UAc for 30s. As an internal standard and to improve the quality of the negative stain, 2ul of tobacco mosaic virus in solution was incubated for 10 seconds on the grid to each sample that did not include AAVs. This step was added after the 2 rounds of washing with H2O and followed by 2 extra H2O washing steps (TMV; kindly supplied by Ruben Diaz-Avalos, New York Structural Biology Center, USA).

Grids were loaded into a Jeol JEM-1400 Plus transmission electron microscope operating a Lab6 electron source at 120 kV. Electron micrographs were recorded on TVIPS XF416 4000 by 4000 pixel charge-coupled device camera (Tietz Video and Image Processing System, Gauting, Germany) at a nominal magnification of 100,000x yielding pictures with a pixel size corresponding to 0.1149 nm at the specimen level.

Images were gaussian blurred 4x in Fiji (78) before being imported into the EMAN2 software package (79). Reference-free alignment was performed on manually selected particles followed by classifications by multivariate statistical analysis. Images were used as indicated without CTF correction or further processing. Particles of interest were manually selected using interactive Particle Picking (e2boxer.py) and subsequently combined into a particle set. Particles were then sorted iteratively in multiple 2D classes using Reference Free Class Averaging (e2refine2d.py) allowing EMAN2 to discard 15% of the picked particles to come to a cleaner result. The center setting was varied, the classaverager was set to mean and default settings were kept for the other parameters. Scale Bars were added using Fiji (78).

### 4.8 Model predictions

Structural model predictions were generated using AlphaFold2 with standard settings (67). To generate the multimeric Fc bound model, two copies of the TRIM21 sequence (full and truncated) were entered as separate molecules and the Fc light and heavy chain were fused into one molecule by adding a long GGGS linker between light and heavy chain. Results were visualized with ChimeraX (80, 81).

## Data availability statement

The original contributions presented in the study are included in the article/**Supplementary Material**. Further inquiries can be directed to the corresponding authors.

## Author contributions

JR: Conceptualization, Data curation, Formal analysis, Investigation, Methodology, Validation, Visualization, Writing – original draft, Writing – review & editing. LF: Writing – original draft, Writing – review & editing, Formal analysis, Investigation, Visualization. JT: Writing – original draft, Investigation. PR: Writing – original draft, Investigation. JB: Writing – original draft, Investigation. TJ: Writing – original draft, Investigation. CK: Writing – review & editing, Conceptualization, Supervision. TS: Writing – review & editing, Conceptualization, Supervision. LL: Writing – review & editing, Conceptualization, Supervision.
