# Supplementary material for: TRIM21 and Fc-engineered antibodies: decoding its complex antibody binding mode with implications for viral neutralization
Source: Front Immunol. 2024 Jun 12;15:1401471. doi: 10.3389/fimmu.2024.1401471 (PMC11210195; doi:10.3389/fimmu.2024.1401471)
Supplement: Supplementary file 1 [file DataSheet_1.docx]

Supplementary Material

| **Sample** | **k_a_**  **(M^-1^s^-1^)** | **Error k_a_**  **(M-1s-1)** | **k_d1_**  **(s^-1^)** | **Error k_d_**  **( s-1)** | **K_D_ (M)** | **Error K_D_ (M)** | **t_1/2_**  **(s)** | **MW Ligand (Da)** | **Ligand Level, R_ligand,exp_ (RU)** | **Ligand Binding Sites / Valency** | **MW Analyte (Da)** | **R_max,exp._ (RU)** | **R_max,theo._ (RU)** | **R_max,Ratio_ (%)** |
| --- | --- | --- | --- | --- | --- | --- | --- | --- | --- | --- | --- | --- | --- | --- |
| mAb1 WT | 2.33E+06 | 5.22E+03 | 9.94E-02 | 2.11E-04 | 4.27E-08 | 1.32E-10 | 7.0 | 146000 | 67.1 | 2 | 25714 | 22.9 | 23.64 | 97 |
| mAb1 WT-AAA | 2.66E+06 | 1.41E+04 | 1.05E-01 | 8.48E-04 | 3.95E-08 | 4.58E-10 | 6.6 | 146000 | 66.9 | 1 | 25714 | 11.2 | 11.78 | 95 |
| mAb1 AAA | NA | NA | NA | NA | NA | NA | NA | NA | NA | NA | NA | NA | NA | NA |

**Table S 1**. Summary of the SPR affinity measurements of immobilized mAb1 Fc variants and TRIM21 PRYSPRY domain in solution using a C1 chip. The kinetic rate parameters are determined from analyzing the sensorgrams shown in Figure 2. The k_ON_, k_OFF_ and K_D_ values are results from a global fit analysis (Langmuir 1:1) ± fitting error.

| (**A**) 10nM PRYSPRY domain | (**B**) 5 nM mAb1 variants | |
| --- | --- | --- |
| **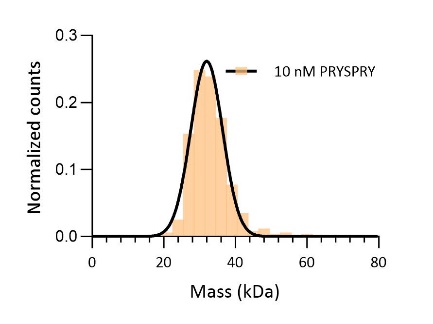** | *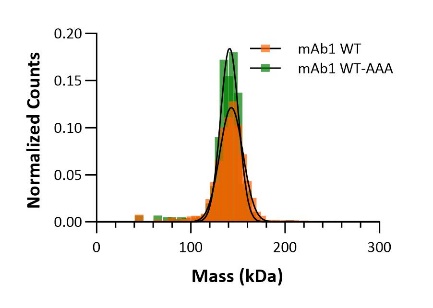* | |
| (**C**) | | |
| **Sample** | **Mass_theo._(kDa)** | **Mass_exp._ (kDa)** |
| (A) PRYSPRY | 25 | 32 ± 5 |
| (B) mAb1 WT | 146 | 143 ± 12 |
| (B) mAb1 WT-AAA | 146 | 141 ± 11 |

**Figure S 1** Mass photometry measurements of individual PRYSPRY domain and mAb1 Fc Variants WT, WT-AAA (**A-C**). Molecular weight determined via mass photometry of individual PRYSPRY domain and mAb1 Fc variants WT, WT-AAA applying Gaussian distribution fit model to measurements. A single PRYSPRY domain appears on the lower limit of detection (25 kDa for Refeyn TwoMP, according to the manufacturer). Results shown in (**C**).

|  | (**A**) Fc WT | | | (**B**) cytokine-Fc Fusion WT | | | (**C**) cytokine-Fc Fusion Y436A | | | (**D**) A20 | | |
| --- | --- | --- | --- | --- | --- | --- | --- | --- | --- | --- | --- | --- |
| **Response (RU)** | **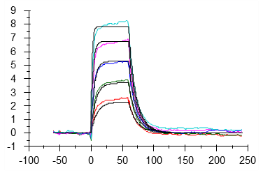** | | | 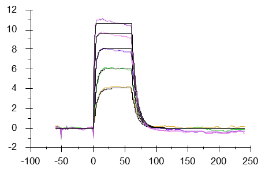 | | | 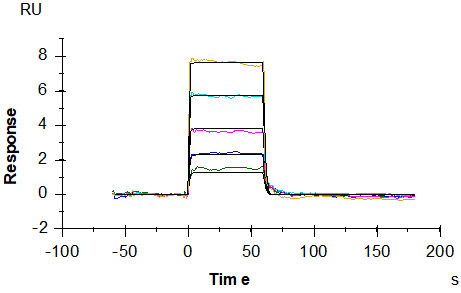 | | | 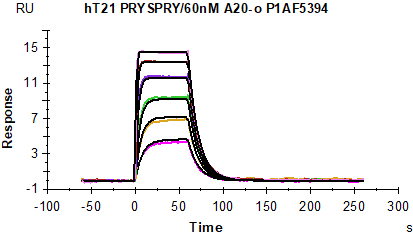 | | |
|  | (**E**) mAb1 WT | | | (**F**) mAb1 WT-AAA | | | (**G**) mAb1 YTE | | | (**H**) mAb1 YTE-AAA | | |
|  | 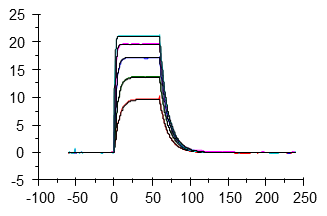 | | | 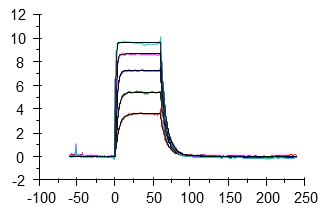 | | | 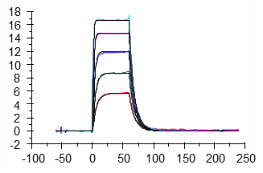 | | | 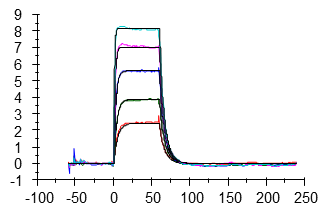 | | |
|  | (**I**) mAb1 HH | | | (**J**) mAb1 HH-AAA | | | (**K**) Briakinumab | | | (L) Ustekinumab | | |
|  | 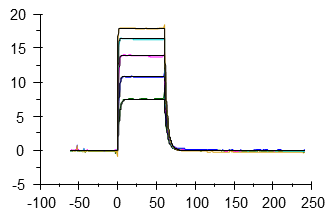 | | | 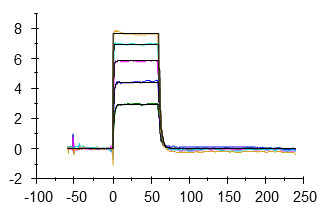 | | | 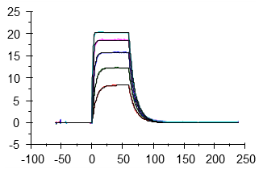 | | | 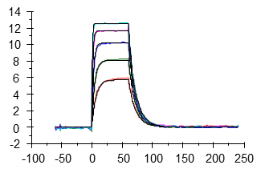 | | |
|  | (**M**) mAb2 WT KiH | | | (**N**) mAb2 WT-AAA | | | (**O**) mAb2 WT | | | (**P**) mAb1 AAA | | |
|  | 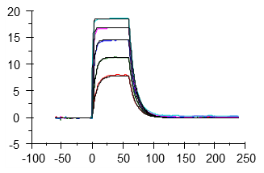 | | | 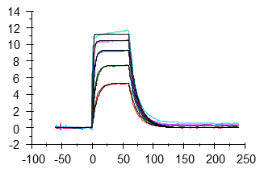 | | | 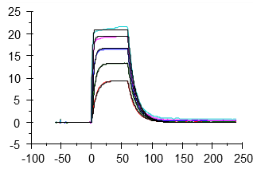 | | | 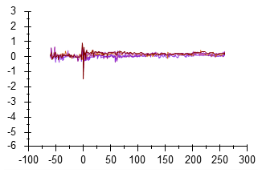 | | |
|  | **Time (s)** | | | | | | | | | | | |
| **(Q)** | | | | | | | | | | | | |
| **Sample** | | **k_ON_**  **(M^-1^s^-1^)** | **Error k_ON_**  **(M-1s-1)** | | **k_OFF_**  **(s^-1^)** | **Error k_OFF_**  **( s-1)** | | **K_D_ (M)** | **Error K_D_**  **(M)** | | **t_1/2_**  **(s)** |  |
| (A) Fc WT | | 1.57E+06 | 1.84E-01 | | 1.02E-01 | 1.40E-03 | | 6.51E-08 | 8.89E-10 | | 6.8 |  |
| (B) cytokine-Fc Fusion WT | | 2.18E+06 | 2.20E+04 | | 1.13E-01 | 1.10E-03 | | 5.19E-08 | 7.27E-10 | | 6.1 |  |
| (C) cytokine-Fc Fusion Y436A | | 2.94E+05 | 2.10E+04 | | 2.25E+00 | 1.60E-01 | | 7.67E-06 | 7.72E-07 | | 0.3 |  |
| (D) A20 | | 1.92E+06 | 8.20E+03 | | 8.76E-02 | 2.60E-04 | | 4.56E-08 | 2.37E-10 | | 7.9 |  |
| (E) mAb1 WT | | 2.33E+06 | 5.22E+03 | | 9.94E-02 | 2.11E-04 | | 4.27E-08 | 1.32E-10 | | 7.0 |  |
| (F) mAb1 WT-AAA | | 2.66E+06 | 1.41E+04 | | 1.05E-01 | 8.48E-04 | | 3.95E-08 | 4.58E-10 | | 6.6 |  |
| (G)mAb1 YTE | | 2.14E+06 | 1.05E+04 | | 1.60E-01 | 7.67E-04 | | 7.48E-08 | 5.13E-10 | | 4.3 |  |
| (H) mAb1 YTE-AAA | | 2.04E+06 | 1.55E+04 | | 1.89E-01 | 1.40E-03 | | 9.26E-08 | 9.83E-10 | | 3.7 |  |
| (I) mAb1 HH | | 4.74E+06 | 4.18E+04 | | 4.77E-01 | 4.17E-03 | | 1.01E-07 | 1.25E-09 | | 1.5 |  |
| (J) mAb1 HH-AAA | | 3.63E+06 | 4.67E+04 | | 4.34E-01 | 5.49E-03 | | 1.20E-07 | 2.16E-09 | | 1.6 |  |
| (K) Briakinumab | | 2.20E+06 | 7.49E+03 | | 1.15E-01 | 3.74E-04 | | 5.23E-08 | 2.46E-10 | | 6.0 |  |
| (L) Ustekinumab | | 2.46E+06 | 1.01E+04 | | 1.04E-01 | 4.08E-04 | | 4.23E-08 | 2.40E-10 | | 6.7 |  |
| (M) mAb2 WT KiH | | 2.38E+06 | 1.11E+04 | | 1.18E-01 | 5.30E-04 | | 4.96E-08 | 3.21E-10 | | 5.9 |  |
| (N) mAb2 WT-AAA | | 2.34E+06 | 2.21E+04 | | 9.01E-02 | 8.12E-04 | | 3.85E-08 | 5.03E-10 | | 7.7 |  |
| (O) mAb2 WT | | 2.09E+06 | 1.49E+04 | | 9.30E-02 | 6.30E-04 | | 4.45E-08 | 4.38E-10 | | 7.5 |  |
| (P) mAb1 AAA | | NA | NA | | NA | NA | | NA | NA | | NA |  |

**Figure S 2** SPR sensorgrams of (antibody) Fc constructs and variants captured onto the biosensor surface (immobilized ligand) and PRYSPRY domain in solution (analyte). PRYSPRY was injected in five different concentration as two-fold dilution series with a highest concentration of (**A-B, D-H, K-O**) 500 nM, (**C+P**) 2000 nM, (**I+J**) 1000 nM. Each plot shows the measured raw data (colored lines) and the global fit analysis as solid lines (black). (**Q**) Summary of the SPR derived kinetic rate parameters of affinity measurements The (antibody) Fc constructs are captured and TRIM21 PRYSPRY domain is in solution. The k_ON_, k_OFF_ and K_D_ values are results from a global fit analysis ± fitting error.

|  | (**A**) Fc WT | | | (**B**) mAb1 WT | | | (**C**) cytokine-Fc Fusion WT | | | (**D**) Briakinumab | |
| --- | --- | --- | --- | --- | --- | --- | --- | --- | --- | --- | --- |
| **Response (RU)** | 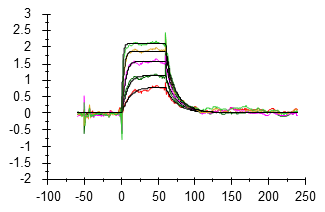 | | | 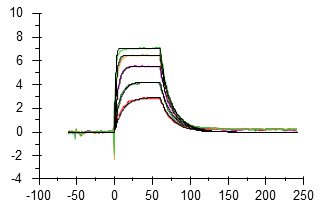 | | | 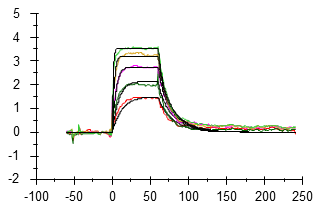 | | | 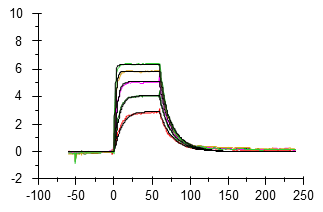 | |
|  | (**E**) Ustekinumab | | | (**F**) mAb2 WT | | |  | | |  | |
|  | 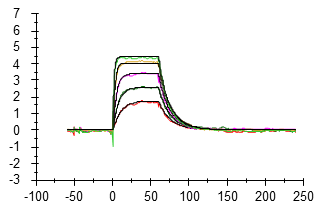 | | | 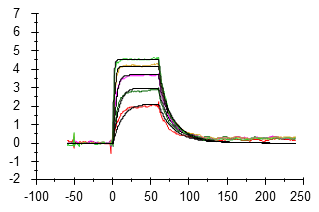 | | |  | | |  | |
|  | **Time (s)** | | | | | | | | | | |
| **(G)** | | | | | | | | | | | |
| **Sample** | | **k_ON_**  **(M^-1^s^-1^)** | **Error k_ON_**  **(M-1s-1)** | | **k_OFF_**  **(s^-1^)** | **Error k_OFF_**  **( s-1)** | | **K_D_ (M)** | **Error K_D_**  **(M)** | | **t_1/2_**  **(s)** |
| (A) Fc WT | | 5.09E+06 | 1.33E+05 | | 1.32E-01 | 3.40E-03 | | 2.59E-08 | 9.52E-10 | | 5.3 |
| (B) mAb1 WT | | 4.36E+06 | 5.31E+04 | | 9.15E-02 | 1.07E-03 | | 2.10E-08 | 3.54E-10 | | 7.6 |
| (C) cytokine-Fc Fusion WT | | 5.88E+06 | 1.57E+05 | | 1.19E-01 | 3.12E-03 | | 2.02E-08 | 7.57E-10 | | 5.8 |
| (D) Briakinumab | | 6.14E+06 | 7.68E+04 | | 1.02E-01 | 1.24E-03 | | 1.66E-08 | 2.90E-10 | | 6.8 |
| (E) Ustekinumab | | 3.89E+06 | 3.76E+04 | | 8.84E-02 | 8.18E-04 | | 2.27E-08 | 3.04E-10 | | 7.8 |
| (F) mAb2 WT | | 7.36E+06 | 1.74E+05 | | 1.20E-01 | 2.78E-03 | | 1.63E-08 | 5.40E-10 | | 5.8 |

**Figure S 3** SPR sensorgrams of PRYSPRY domain immobilized onto the biosensor surface (ligand) and (antibody) Fc constructs and variants in solution (analyte). Fc variants were injected in five different concentrations as two-fold dilution series with a highest concentration of (A-F) 200 nM. Each plot shows the measured raw data (coloured lines) and the global fit analysis as solid lines (black). The interaction is described by a monophasic fit model reflecting the affinity binding mode. (G) Summary of the SPR derived kinetic rate parameters of affinity measurements. TRIM21 PRYSPRY domain is immobilized and (antibody) Fc constructs are in solution. The k_ON_, k_OFF_ and K_D_ values are results from a global fit analysis ± fitting error.

| (**A**) | | | | | | | | | | | | | |
| --- | --- | --- | --- | --- | --- | --- | --- | --- | --- | --- | --- | --- | --- |
|  | |  | | **TRIM21(T21)-CC-PS + mAb1 Fc variants: Molar Ratios** | | | | | | | | | |
|  |  |  |  | | mAb1 WT | | | mAb1 WT-AAA | | | mAb1 AAA | | |
|  | Molar Ratio  T21-CC-PS:mAb1 | Species | Mass_exp._  (kDa) | | Mean (kDa) | SD (kDa) | Gaussian Fit (%) | Mean (kDa) | SD (kDa) | Gaussian Fit (%) | Mean (kDa) | SD (kDa) | Gaussian Fit (%) |
| T21-CC-PS  2.5 nM & mAb1 7.5 nM | 1:3 | T21 | 86 | | 75 | 12.6 | 3 | 76 | 14.9 | 6 | 77 | 17.9 | 3 |
|  |  | IgG | 146 | | 144 | 12.9 | 93 | 144 | 13.8 | 90 | 141 | 13.9 | 94 |
|  |  | T21-IgG | 232 | | 228 | 40 | NA | NA | NA | NA | NA | NA | NA |
| T21-CC-PS 5 nM & mAb1 5 nM | 1:1 | T21 | 86 | | 74 | 16.8 | 10 | 75 | 15 | 16 | 75 | 16 | 18 |
|  |  | IgG | 146 | | 148 | 13.2 | 78 | 145 | 12.3 | 80 | 144 | 12.5 | 80 |
|  |  | T21-IgG | 232 | | 236 | 13.8 | 7 |  |  | NA | NA | NA | NA |
| T21-CC-PS 7.5 nM & mAb1 2.5 nM | 3:1 | T21 | 86 | | 76 | 12.4 | 31 | 76 | 13.9 | 33 |  |  |  |
|  |  | IgG | 146 | | 141 | 11.2 | 49 | 145 | 14.1 | 64 |  |  |  |
|  |  | T21-IgG | 232 | | 231 | 12.7 | 15 | NA | NA | NA |  |  |  |
| T21-CC-PS 22.5 nM & mAb1 2.5 nM | 10:1 | T21 | 86 | | 75 | 14.5 | 62 | 75 | 14.9 | 66 | 75 | 14.1 | 66 |
|  |  | IgG | 146 | | 138 | 18.5 | 8 | 144 | 26 | 31 | 141 | 23 | 31 |
|  |  | T21-IgG | 232 | | 233 | 16.7 | 24 | NA | NA | NA | NA | NA | NA |
|  | | | | | | | | | | | | | |
| (**B**) | | | | | | | | | | | | | |
| 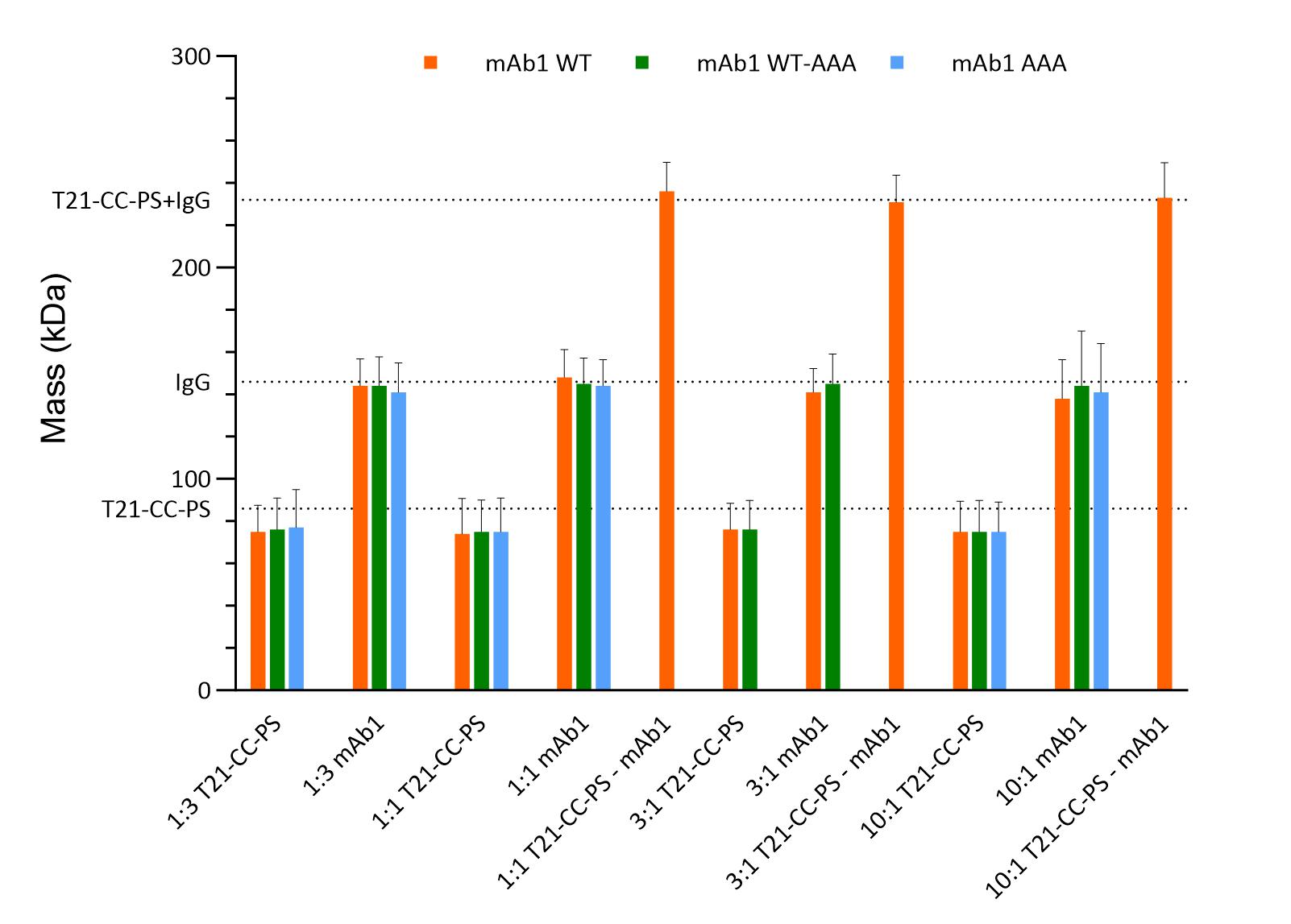 | | | | | | | | | | | | | |

**Figure S 4** Molecular weight determined via mass photometry of TRIM21-CC-PS complexed with mAb1 Fc variants WT, WT-AAA and AAA applying Gaussian Distribution Fit Model (**A**). (**B**) Data from (A) is visualized applying Gaussian Distribution Fit Model over the applied stoichiometric ratios.

|  | (**A**) mAb1 WT | | | | | | | **(B**) mAb1 WT_Assoc. | | | | | | (**C**) mAb1 YTE | | | | | | |
| --- | --- | --- | --- | --- | --- | --- | --- | --- | --- | --- | --- | --- | --- | --- | --- | --- | --- | --- | --- | --- |
| **Response (RU)** | 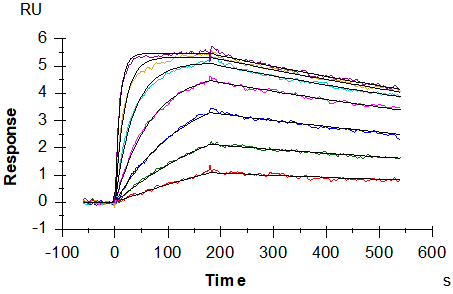 | | | | | | | 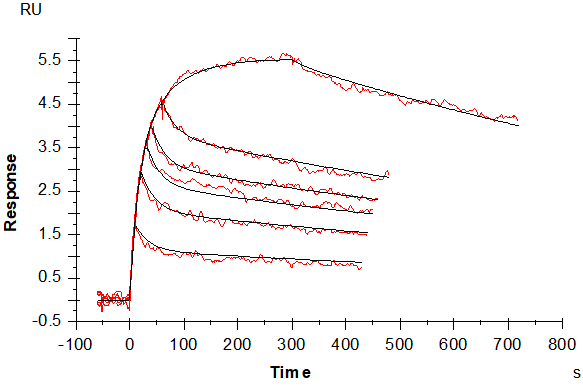 | | | | | | 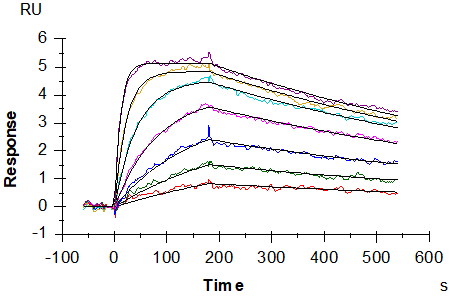 | | | | | | |
|  | (**D**) mAb1 HH | | | | | | | (**E**) cytokine-Fc Fusion Y436A | | | | | |  | | | | | | |
|  | 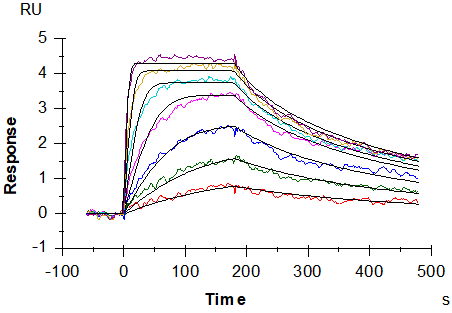 | | | | | | | 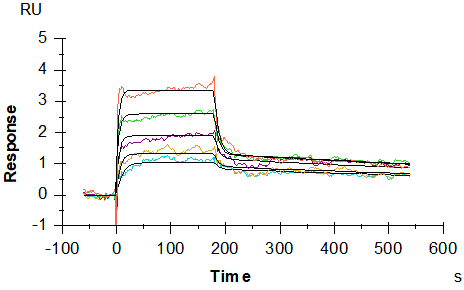 | | | | | |  | | | | | | |
|  | (**F**) mAb1 WT-AAA | | | | | | | (**G**) mAb1 YTE-AAA | | | | | | (**H**) mAb1 HH-AAA | | | | | | |
|  | 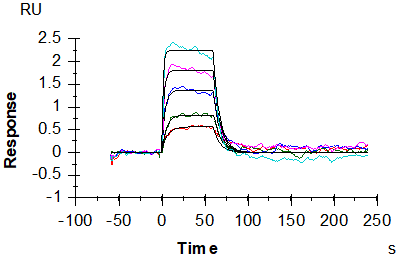 | | | | | | | 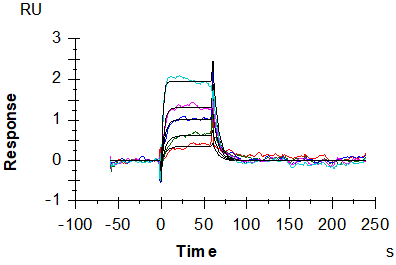 | | | | | | 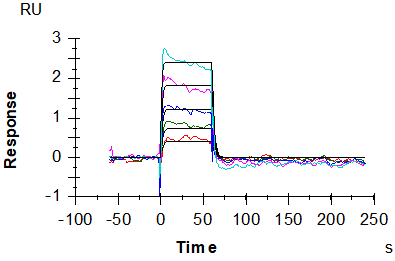 | | | | | | |
|  | **Time (s)** | | | | | | | | | | | | | | | | | | | |
| (**I**) | | | | | | | | | | | | | | | | | | | | |
| **Sample** | | | | **k_ON_**  **(M^-1^s^-1^)** | | **Error k_ON_**  **(M-1s-1)** | | | | **k_OFF_**  **(s^-1^)** | | **Error k_OFF_**  **( s-1)** | | | **K_D_ (M)** | | **Error K_D_**  **(M)** | | | **t_1/2_**  **(s)** |
| (**A**) TRIM21-CC-PS - mAb1 WT | | | | 1.11E+06 | | 1.30E+03 | | | | 7.62E-04 | | 9.30E-07 | | | 6.873E-10 | | 1.16E-12 | | | 910.2 |
| (**C**) TRIM21-CC-PS - mab1 YTE | | | | 8.65E+05 | | 1.40E+03 | | | | 1.27E-03 | | 1.50E-06 | | | 1.466E-09 | | 2.94E-12 | | | 546.6 |
| (**D**) TRIM21-CC-PS - mAb1 HH | | | | 4.74E+06 | | 3.90E+04 | | | | 6.51E-03 | | 4.00E-05 | | | 1.374E-09 | | 1.41E-11 | | | 106.5 |
| (**E**) TRIM21-CC-PS - cytokine-Fc Fusion Y436A | | | | 2.46E+05 | | 3.70E+03 | | | | 0.132 | | 0.0012 | | | 5.359E-07 | | 9.41E-09 | | | 5.3 |
| (**F**) TRIM21-CC-PS - mAb1 WT-AAA | | | | 1.10E+06 | | 2.00E+04 | | | | 0.1285 | | 0.0013 | | | 1.165E-07 | | 2.42E-09 | | | 5.4 |
| (**G**) TRIM21-CC-PS - mab1 YTE-AAA | | | | 7.58E+05 | | 1.40E+04 | | | | 0.1669 | | 0.0015 | | | 2.201E-07 | | 4.52E-09 | | | 4.2 |
| (**H**) TRIM21-CC-PS - mAb1 HH-AAA | | | | 9.21E+05 | | 2.10E+04 | | | | 0.4367 | | 0.0085 | | | 4.742E-07 | | 1.42E-08 | | | 1.6 |
|  | | | | | | | | | | | | | | | | | | | | |
| (**J**) | | | | | | | | | | | | | | | | | | | | |
| **Sample** | | k_ON1_  (M^-1^s^-1^) | Error k_ON1_  (M-1s-1) | | k_OFF1_  ( s^-1^) | | Error k_OFF1_  ( s-1) | | k_ON2_  (s^-1^) | | Error k_ON2_  (s-1) | | k_OFF2_  (s^-1^) | | | Error k_OFF2_  ( s-1) | | **K_D_ (M)** | Error K_D_ (M) | |
| (**B**) mAb1 WT_Assoc | | 2.04E+06 | 1.40E+04 | | 0.01885 | | 1.90E-04 | | 0.02228 | | 1.10E-04 | | 0.001669 | | | 6.80E-06 | | **6.44E-10** | 1.13E-10 | |

**Figure S 5** SPR sensorgrams of (antibody) Fc variants (symmetric and asymmetric) immobilized onto the biosensor surface (ligand) and TRIM21-CC-PS in solution (analyte). TRIM21-CC-PS was injected in several different concentrations as two-fold dilution series with a highest concentration of (**A, C-D**) 100 nM, (**B**) 25 nM, (**E**) 400 nM, (**F-G**) 500 nM and (**H**) 1000 nM. Each plot shows the measured raw data (coloured lines) and the global fit analysis as solid lines (black). The interaction is described by a monophasic fit model, reflecting the affinity (**F - H**) or avidity (**A –E**) binding mode, expected for (**B**) where the two state model was applied. (**I-J**) Summary of the SPR derived kinetic rate parameters of affinity and avidity measurements.(**I**) The k_ON_, k_OFF_ and K_D_ values are results from a global fit analysis ± fitting error (1:1 Langmuir Fit). (**J**) 25nM of TRIM21-CC-PS are injected with different association times (10 sec - 300 sec). The k_ON_, k_OFF_ and K_D_ values are results from a global fit analysis ± fitting error (Two State Model).

|  | (**A**) 5nM mAb2 WT | |
| --- | --- | --- |
|  | 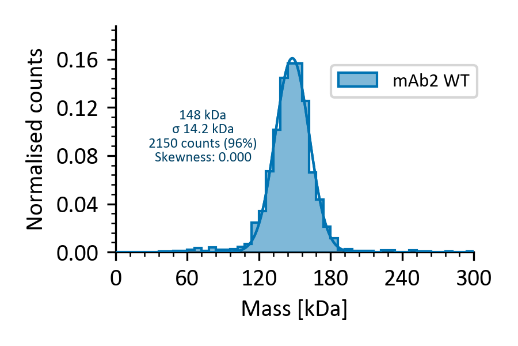 | |
| (**B**) Fc WT / cytokine-Fc Fusion WT / cytokine-Fc Fusion WT-AAA (equivalent mass to cytokine-Fc Fusion Y436A) | (**C**) mAb1 WT / YTE / HH | |
| 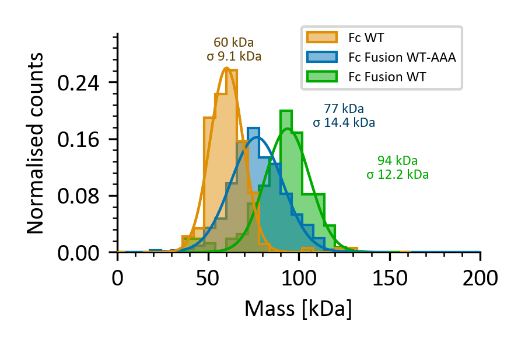 | 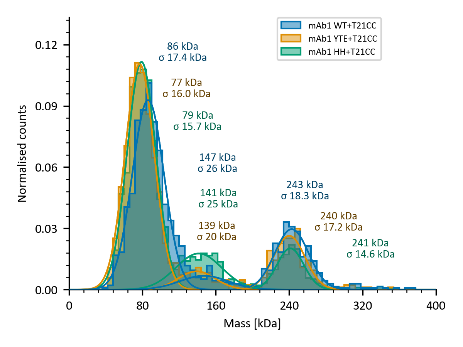 |  |
| (**D**) mAb 2 WT / WT-AAA | (**E**) Fc WT / WT-AAA | |
| 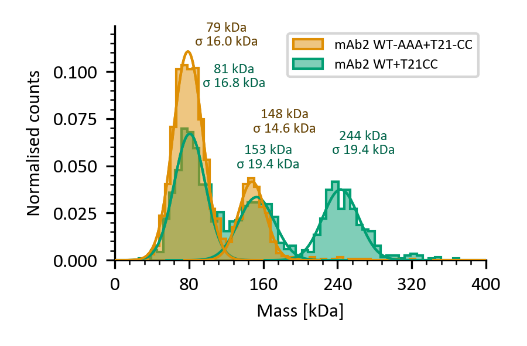 | 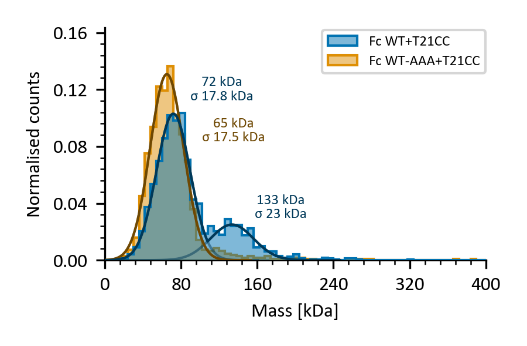 | |
| (**F**) cytokine-Fc Fusion WT / WT-AAA / Y436A | (**G**) mAb1 YTE-AAA / HH-AAA | |
| 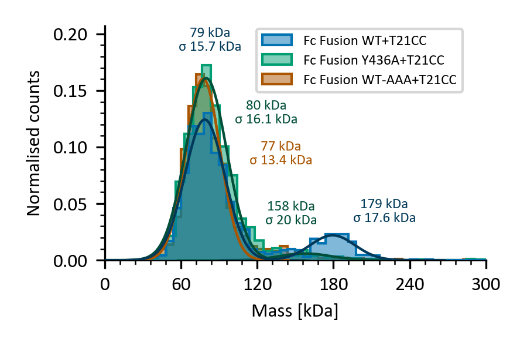 | 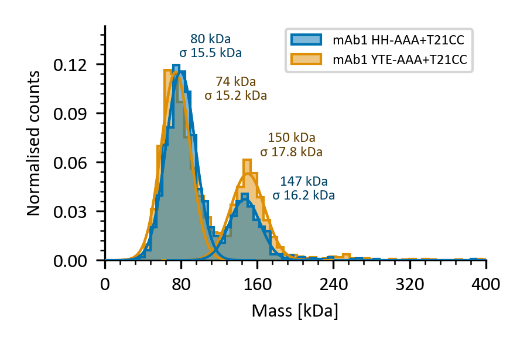 | |
| (**H**) Briakinumab / Ustekinumab |  | |
| 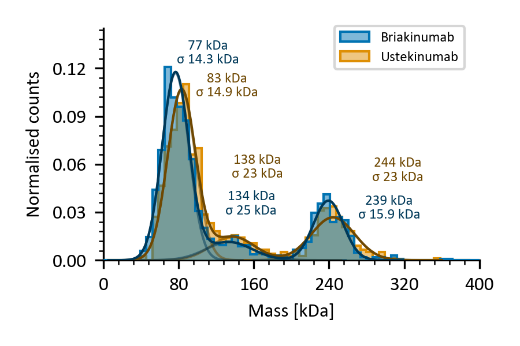 |  | |

**Figure S 6** Molecular weight determined via mass photometry of TRIM21-CC-PS complexed with (antibody) Fc variants applying Gaussian Distribution Fit Model to the occurring species. 2.5 nM Fc variants are measured in the presence of 22.5 nM TRIM21-CC-PS using MP One device. Data is shown in **Table S 2**.

| **Controls / Individual Mass** | | | | | | | |
| --- | --- | --- | --- | --- | --- | --- | --- |
| **Sample** | | **Mass_theo._(kDa)** | | **Mass_exp._ (kDa)** | | **Gaussian Fit (%)** | |
| (**A**) 5 nM mAb2 WT | | 146 | | 148 ± 114 | | 96 | |
| (**B**) Fc WT | | 52 | | 60 ± 9 | | 100 | |
| (**B**) cytokine-Fc Fusion WT-AAA (equals cytokine-Fc Fusion Y436A in mass) | | 71 | | 77 ± 14 | | 98 | |
| (**B**) Fc Fusion WT | | 91 | | 94 ± 12 | | 93 | |
|  | | | | | | | |
| **TRIM21-CC-PS (22.5nM) + Fc variants (2.5 nM): Molar Ratio 10:1** | | | | | | | |
| **Sample: TRIM21-CC-PS +** |  | | **Species** | | | | |
|  |  | | TRIM21-CC-PS | | Fc variant | | TRIM21-CC-PS  + Fc variant |
| **(C) mAb1 WT** | | | | | | | |
| Mass_theo._ (kDa) |  | | 86 | | 146 | | 232 |
| Mass_exp._ (kDa) ± SD |  | | 86 ± 17 | | 147 ± 26 | | 243 ± 18 |
| Gaussian Fit (%) |  | | 69 | | 8 | | 23 |
| **(C) mAb1 YTE** | | | | | | | |
| Mass_theo._ (kDa) |  | | 86 | | 146 | | 232 |
| Mass_exp._ (kDa) ± SD |  | | 77 ± 16 | | 139 ± 20 | | 240 ± 17 |
| Gaussian Fit (%) |  | | 74 | | 8 | | 19 |
| **(C) mAb1 HH** | | | | | | | |
| Mass_theo._ (kDa) |  | | 86 | | 146 | | 232 |
| Mass_exp._ (kDa) ± SD |  | | 79 ± 16 | | 141 ± 25 | | 241 ± 15 |
| Gaussian Fit (%) |  | | 73 | | 19 | | 12 |
| **(D) mAb2 WT** | | | | | | | |
| Mass_theo._ (kDa) |  | | 86 | | 146 | | 232 |
| Mass_exp._ (kDa) ± SD |  | | 81 ± 17 | | 153 ± 20 | | 244 ± 20 |
| Gaussian Fit (%) |  | | 44 | | 25 | | 28 |
| **(D) mAb2 WT-AAA** | | | | | | | |
| Mass_theo._ (kDa) |  | | 86 | | 146 | | 232 |
| Mass_exp._ (kDa) ± SD |  | | 79 ± 16 | | 148 ± 15 | | NA |
| Gaussian Fit (%) |  | | 74 | | 25 | | NA |
| **(E) Fc WT** | | | | | | | |
| Mass_theo._ (kDa) |  | | 86 | | 52 | | 138 |
| Mass_exp._ (kDa) ± SD |  | | 72 ± 18 (overlapping) | | | | 133 ± 23 |
| Gaussian Fit (%) |  | | 77 (overlapping) | | | | 24 |
| **(E) Fc WT-AAA** | | | | | | | |
| Mass_theo._ (kDa) |  | | 86 | | 52 | | 138 |
| Mass_exp._ (kDa) ± SD |  | | 65 ± 18 (overlapping) | | | | NA |
| Gaussian Fit (%) |  | | 96 (overlapping) | | | | NA |
| **(F) cytokine-Fc Fusion WT** | | | | | | | |
| Mass_theo._ (kDa) |  | | 86 | | 91 | | 177 |
| Mass_exp._ (kDa) ± SD |  | | 79 ± 16 (overlapping) | | | | 179 ± 18 |
| Gaussian Fit (%) |  | | 82 (overlapping) | | | | 16 |
| **(F) cytokine-Fc Fusion Y436A** | | | | | | | |
| Mass_theo._ (kDa) |  | | 86 | | 71 | | 157 |
| Mass_exp._ (kDa) ± SD |  | | 80 ± 16 (overlapping) | | | | 158 ± 20 |
| Gaussian Fit (%) |  | | 95 (overlapping) | | | | 4 |
| **(F) cytokine-Fc Fusion WT-AAA** | | | | | | | |
| Mass_theo._ (kDa) |  | | 86 | | 71 | | 157 |
| Mass_exp._ (kDa) ± SD |  | | 77 ± 14 (overlapping) | | | | NA |
| Gaussian Fit (%) |  | | 90 (overlapping) | | | | NA |
| **(G) mAb1 YTE-AAA** | | | | | | | |
| Mass_theo._ (kDa) |  | | 86 | | 146 | | 157 |
| Mass_exp._ (kDa) ± SD |  | |  | |  | | NA |
| Gaussian Fit (%) |  | |  | |  | | NA |
| **(G) mAb1 HH-AAA** | | | | | | | |
| Mass_theo._ (kDa) |  | | 86 | | 146 | | 232 |
| Mass_exp._ (kDa) ± SD |  | | 74 ± 15 | | 150 ± 18 | | NA |
| Gaussian Fit (%) |  | | 63 | | 34 | | NA |
| **(H) BriakinumAb** | | | | | | | |
| Mass_theo._ (kDa) |  | | 86 | | 146 | | 232 |
| Mass_exp._ (kDa) ± SD |  | | 77 ± 14 | | 134 ± 25 | | 239 ± 16 |
| Gaussian Fit (%) |  | | 64 | | 11 | | 23 |
| **(H) UstekinumAb** | | | | | | | |
| Mass_theo._ (kDa) |  | | 86 | | 146 | | 232 |
| Mass_exp._ (kDa) ± SD |  | | 83 ± 15 | | 138 ± 23 | | 244 ± 23 |
| Gaussian Fit (%) |  | | 62 | | 13 | | 24 |

**Table S 2** Molecular weight determined via mass photometry of TRIM21-CC-PS complexed with (antibody) Fc variants applying Gaussian Distribution Fit Model. The mass photometry data was analyzed using the DiscoverMP 2.5.0 software.

| (**A**) A20 WT | | | | | (**B**) A20 half WT | | | | | (**C**) A20 half WT-AAA | | | | |
| --- | --- | --- | --- | --- | --- | --- | --- | --- | --- | --- | --- | --- | --- | --- |
| 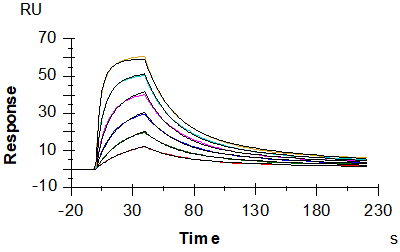 | | | | | 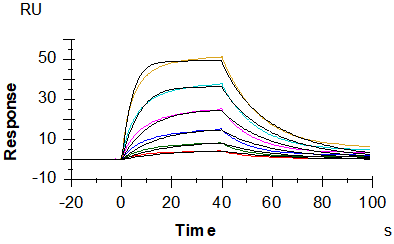 | | | | | 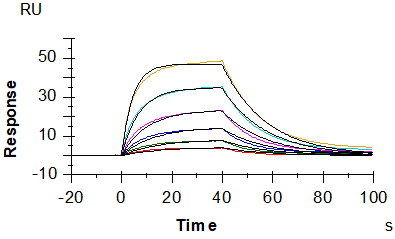 | | | | |
| (**D**) | | | | |  | | | | |  | | | | |
| **Sample** | k_ON1_  (M^-1^s^-1^) | Error k_ON1_  (M^-1^s^-1^) | k_OFF1_ (s^-1^) | Error  k_OFF1_ (s^-1^) | | k_ON2_  (s^-1^) | Error k_ON2_  (s^-1^) | k_OFF2_  (s^-1^) | Error  k_OFF2_  (s^-1^) | | K_D1_ (M) | Error K_D1_ (M) | K_D2_ (M) | Error K_D2_ (M) |
| (**A**) A20 WT | 5.91E+05 | 3.50E+03 | 0.04481 | 1.10E-04 | | 1.46E+06 | 3.00E+03 | 0.007248 | 2.90E-05 | | 7.59E-08 | 4.87E-10 | 4.96E-09 | 2.23E-11 |
| (**B**) A20 half WT | 7.33E+05 | 1.20E+04 | 0.06817 | 9.30E-04 | |  |  |  |  | | 9.31E-08 | 1.98E-09 |  |  |
| (**C**) A20 half WT-AAA | 4.16E+05 | 2.50E+03 | 0.05738 | 1.00E-04 | |  |  |  |  | | 1.38E-07 | 8.63E-10 |  |  |

**Figure S 7** SPR sensorgrams of AAV2 immobilized (standard amine coupling) onto the biosensor surface and A20 antibody variants in solution. A20 variants were injected in six different concentrations as two-fold dilution series with a highest concentration of (**A**) 200 nM and (**B-C**) 400 nM. Each plot shows the measured raw data (colored lines) and the global fit analysis as solid lines (black). The interaction for (**B-C**) is described by a monophasic fit model, reflecting the affinity-binding mode. (**A**) is a bivalent analyte (A20 WT) and can be described by a heterogeneous model reflecting affinity and avidity. Kinetic rate parameters are displayed in (**D**). The k_ON_, k_OFF_ and K_D_ values are results from a global fit analysis ± fitting error (Heterogeneous model (**A**) and 1:1 Langmuir Fit (**B-C**)).

| (**A**) A20 WT / TRIM21-CC-PS | | | | | | | |
| --- | --- | --- | --- | --- | --- | --- | --- |
| 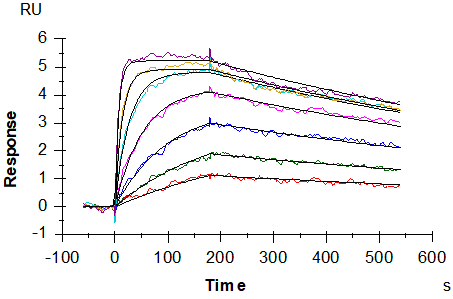 | | | | | | | |
| (**B**) | | | | | | | |
| **Sample** | k_ON_  (M^-1^s^-1^) | Error k_ON_  (M^-1^s^-1^) | k_OFF_  (s^-1^) | Error k_OFF_  (s^-1^) | K_D_ (M) | Error K_D_  (M) | t_1/2_  (s) |
| TRIM21-CC-PS – A20 WT | 1.44E+06 | 3.40E+03 | 9.83E-04 | 1.50E-06 | 6.847E-10 | 1.93E-12 | 705.4 |

**Figure S 8** SPR sensorgrams of A20 WT immobilized onto the biosensor surface (ligand) and TRIM21-CC-PS in solution (analyte). (**A**) TRIM21-CC-PS was injected in several different concentrations as two-fold dilution series with a highest concentration of 100 nM. Each plot shows the measured raw data (coloured lines) and the global fit analysis as solid lines (black). (**B**) The interaction is described by a monophasic fit model, reflecting the avidity-binding mode. The k_ON_, k_OFF_ and K_D_ values are results from a global fit analysis ± fitting error (1:1 Langmuir Fit).

|  | **Low A20 Density** | **High A20 Density** | |
| --- | --- | --- | --- |
|  | (**A**) A20 WT | (**B**) A20 WT | |
| **Response (RU)** | 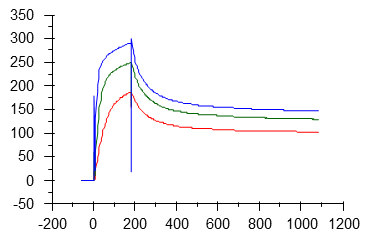 | 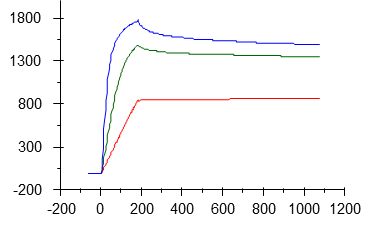 | |
|  | (**C**) A20 half WT | (**D**) A20 half WT | |
|  | 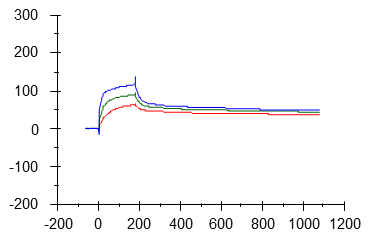 | 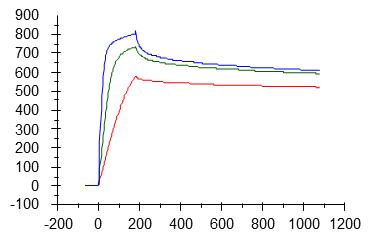 |  |
|  | (**E**) A20 half WT-AAA | (**F**) A20 half WT-AAA | |
|  | 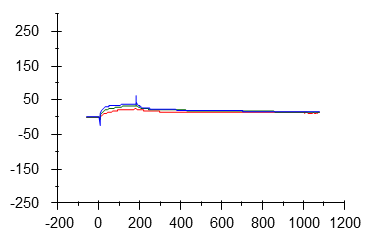 | 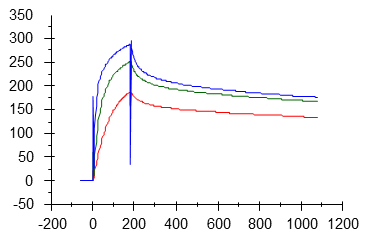 | |
|  | **Time (s)** | | |

**Figure S 9** SPR Sensorgrams showing AAV2 binding to captured anti-capsid antibody A20 variants on a low or high (8x) TRIM21-CC-PS ligand density. Injections of 3 concentrations of rAAVv-2 3-fold dilution with a highest concentration of 3.32 nM.


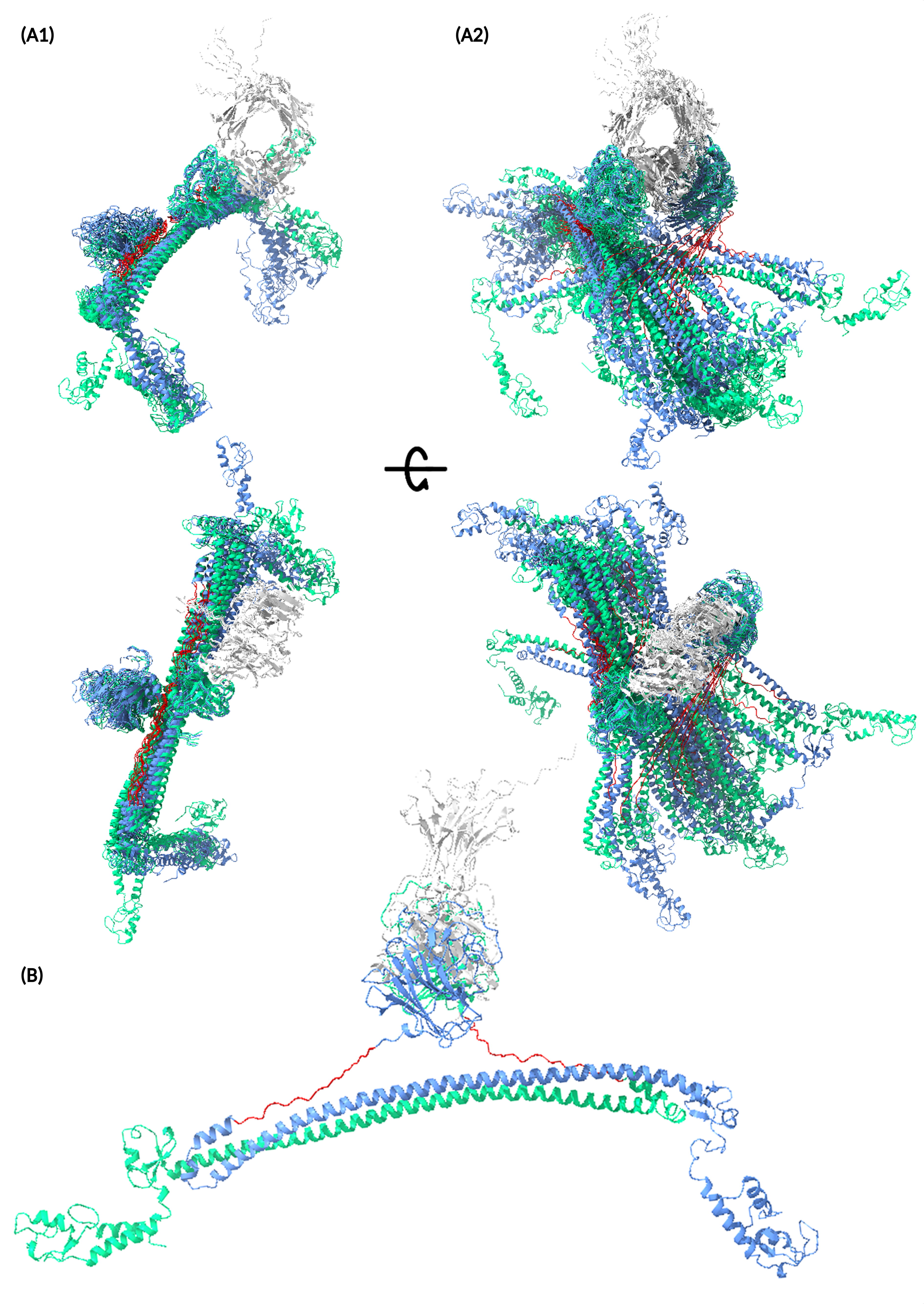


**Figure S 10** Model predictions of TRIM21 and mode of action (PDB-ID: 2IWG, Uniprot ID P19474). (**A-B**) Structural predictions of alphafold of 2 copies of full length TRIM21 as well as an Fc domain. A minority of predictions maintain the association of the PRYSPRY domain with the coiled coil domain (**A1**) whereas the majority of structures release this association (**A2**). Variations in structural predictions for the coiled-coil domain are much higher when the PRYSPRY domain is not associating with it and the RBCC predictions vary highly after binding the Fc domain in both model variants. (**B**) Highlighted prediction of Alphafold2 for the TRIM21 - Fc complex showing dislodged PRYSPRY as well as variation in RB domain.
